# Deubiquitinase USP17 negatively regulates 3T3-L1 adipocyte differentiation via HDAC1

**DOI:** 10.1016/j.gendis.2025.101930

**Published:** 2025-11-12

**Authors:** Yuankuan Li, Meiyu Piao, Yujian Jin, Sung Ho Lee, Kwang Youl Lee

**Affiliations:** a College of Pharmacy, Research Institute of Pharmaceutical Sciences, Chonnam National University, Gwangju 61186, South Korea; b Department of Biomedical Laboratory Science, Gwangju Health University, Gwangju 62287, South Korea

According to the World Health Organization (WHO), approximately 890 million people in the world were living with obesity in 2022. In South Korea, the prevalence of obesity has gradually increased, rising from 30.2% to 38.4% (2012–2021).^1^ Ubiquitin-specific protease 17 (USP17) significantly impacts critical cellular processes, including cell proliferation and oncogenesis.^2^ However, the role of USP17 in adipogenesis, a key process in obesity development, remains incompletely understood. Here, we investigated the functions of USP17 and the underlying mechanisms in 3T3-L1 adipocyte differentiation. Notably, a remarkable reduction in USP17 expression was observed following the induction of differentiation, largely due to dexamethasone. We then identified USP17 as a negative regulator of adipogenesis, as indicated by reduced lipid accumulation and down-regulation of adipogenic genes. Furthermore, USP17's deubiquitinating activity was indispensable for its regulation of adipocyte differentiation. Moreover, we identified histone deacetylase 1 (HDAC1) as a direct substrate of USP17. By stabilizing HDAC1 through deubiquitination, wild-type USP17, but not the catalytically inactive mutant USP17 (C89S), suppresses the transcriptional activity of peroxisome proliferator-activated receptor gamma (PPARγ). However, HDAC1 was found to partially counteract the resulting increase in PPARγ activity induced by USP17 (C89S). Overall, our findings highlight the potential of USP17 as a promising therapeutic target for obesity therapy.

In our previous work, we demonstrated that USP17 could promote osteoblast differentiation in C2C12 cells.^2^ Given that osteoblast differentiation and adipocyte differentiation are reciprocally regulated processes, we speculated that USP17 may also be involved in regulating the latter. We first examined the expression of USP17 during 3T3-L1 adipocyte differentiation. *Usp1*7 mRNA levels significantly decreased at the early stages, coinciding with the peak expression of CCAAT enhancer binding protein beta (C/EBPβ), an early adipogenic marker, and the gradual increase in late-stage markers C/EBPα and PPARγ (Fig. 1A; Fig. S1A–S1C). Considering that 3T3-L1 adipocyte differentiation is initiated by the differentiation cocktail comprising 3-Isobutyl-1-methylxanthine (IBMX), dexamethasone (Dex), and insulin, we aimed to identify which component was dominantly responsible for the observed reduction in *Usp17* expression. We observed that IBMX slightly reduced the mRNA level of *Usp17* while significantly increasing *Cebpb* expression. Insulin markedly increased *Usp17* expression, peaking at 24 h post-treatment before declining. Notably, only Dex displayed a pattern consistent with that of the differentiation cocktail in terms of *Usp17* expression (Fig. 1B and C). Moreover, *Usp17* expression decreased in both time- and dose-dependent manners with Dex treatment, reaching its lowest level at 12 h post-treatment (Fig. S1D and S1E). These results indicate that among the components of the differentiation cocktail, Dex is responsible for the reduction in *Usp17* expression during 3T3-L1 adipocyte differentiation.

**Figure 1:**
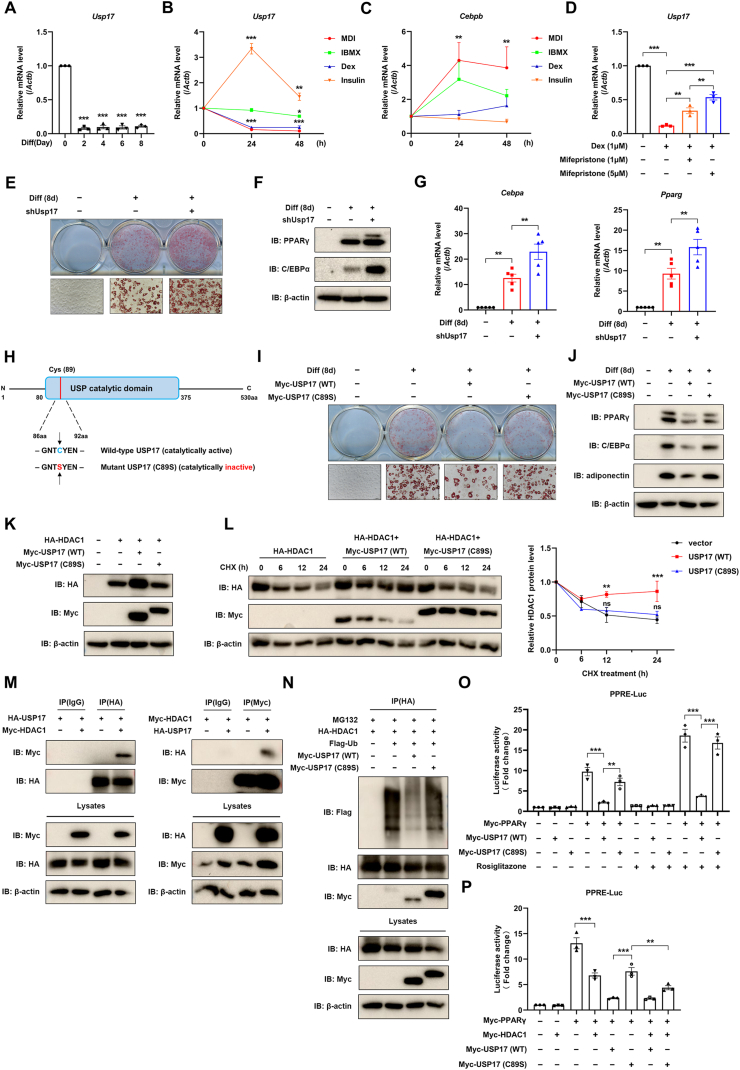
USP17 acts as a negative regulator of 3T3-L1 adipocyte differentiation via HDAC1. After transfection, the two-day growth-arrested 3T3-L1 cells were primed for an eight-day adipogenic differentiation using the described differentiation cocktail. **(A)** The mRNA levels of *Usp17* were measured in 3T3-L1 cells at different timepoints (days 0, 2, 4, 6, and 8). **(B, C)** The mRNA levels of *Usp17* and *Cebpb* were measured in 3T3-L1 cells (48 h post–confluence) treated with MDI or its components (1 μM Dex, 5 μg/mL insulin, and 0.5 mM IBMX). **(D)** The effect of mifepristone on Dex-induced decrease in *Usp1*7 mRNA levels. Quantitative reverse transcription PCR was conducted to evaluate the mRNA levels, and *Actb* was used as the loading control. The results were shown as mean ± standard error of the mean. ∗*P* < 0.05, ∗∗*P* < 0.01, and ∗∗∗*P* < 0.001. **(E)** Representative images of differentiated 3T3-L1 cells, with or without *Usp17* knockdown, at 200 × magnification. The scale bar represents 100 μm. **(F)** The impact of *Usp17* knockdown on PPARγ and C/EBPα protein levels in differentiated 3T3-L1 cells was measured by immunoblotting. **(G)** The effect of *Usp17* knockdown on the mRNA levels of *Cebpa* and *Pparg* in differentiated 3T3-L1 cells was assessed using quantitative reverse transcription PCR. **(H)** A schematic representation depicting the substitution of Cys (C) at position 89 with Ser (S) in USP17, resulting in a catalytically inactive mutant. **(I)** Representative images of differentiated 3T3-L1 cells, with or without the overexpression of wild-type USP17 (WT) or the catalytically inactive mutant USP17 (C89S), at 200 × magnification. The scale bar represents 100 μm. **(J)** The effects of USP17's catalytic activity on the protein levels of PPARγ, C/EBPα, and adiponectin were measured by immunoblotting. **(K)** To assess whether USP17's catalytic activity is required for HDAC1 stabilization, HA-HDAC1 and either wild-type Myc-USP17 (WT) or the catalytically inactive mutant Myc-USP17 (C89S) were co-transfected into HEK 293T cells. **(L)** HDAC1's half-life was evaluated by treating the transfected HEK 293T cells with 50 μg/mL CHX for varying durations (0, 6, 12, and 24 h), followed by immunoblotting and quantification. **(M)** Immunoprecipitation with the control IgG, anti-HA, and anti-Myc antibodies was used to determine the reciprocal interactions between HA-tagged USP17 and Myc-tagged HDAC1. **(N)** Ubiquitination of HDAC1 was assessed in HEK 293T cells. The cells were transfected with HA-HDAC1, Flag-tagged ubiquitin (Flag-Ub), and either Myc-USP17 (WT) or Myc-USP17 (C89S) for 24 h and then treated with 10 μM MG132 for 8 h. **(O)** Luciferase reporter assay was used to examine the effects of wild-type USP17 and USP17 (C89S) on the transcriptional activities of the luciferase reporter plasmid (PPRE-Luc), which features the consensus PPAR responsive element (PPRE). **(P)** HDAC1 diminished the positive effect of USP17 (C89S) on the transcriptional activities of PPRE-Luc. Luciferase activity is represented as fold changes in comparison to the control group. The results were shown as mean ± standard error of the mean. ∗∗*P* < 0.01 and ∗∗∗*P* < 0.001. Dex, dexamethasone; IBMX, 3-Isobutyl-1-methylxanthine; MDI; a differentiation cocktail comprising Dex, insulin, and IBMX; CHX, cycloheximide; PPRE, PPAR responsive element.

Dex is a glucocorticoid that binds to the glucocorticoid receptor (GR) in the cytoplasm. Thus, we investigated the involvement of GR in the Dex-induced reduction in *Usp17* expression. This reduction was attenuated by mifepristone, a potent GR antagonist, in a dose-dependent manner (Fig. 1D). To further explore this trend, we transfected 3T3-L1 preadipocytes with a shRNA targeting *Nr3c1*, the GR-encoding gene. In the absence of Dex, *Nr3c1* knockdown did not affect *Usp17* expression. However, in the presence of Dex, *Nr3c1* knockdown partially reversed its expression, albeit to a lesser extent due to the low knockdown efficacy (Fig. S1F and S1G). Additionally, Dex treatment down-regulated interleukin-6 (IL-6), mirroring the pattern of Dex-induced USP17 expression (Fig. S1H). Mifepristone also dose-dependently alleviated the decrease in IL-6 expression caused by Dex and *Nr3c1* knockdown and elevated its level regardless of Dex presence (Fig. S1I and S1J), suggesting GR is an upstream regulator of IL-6. Given that IL-6 is one of the cytokines to induce USP17 expression,^3^ a plausible hypothesis is that the Dex-induced reduction in *Usp17* expression is mediated by the GR/IL-6 signaling axis.

To elucidate the functional importance of USP17 in 3T3-L1 adipocyte differentiation, we transfected 3T3-L1 cells with shRNA targeting *Usp17*. Knockdown of *Usp17* enhanced lipid droplet accumulation and elevated intracellular triglyceride levels (Fig. 1E; Fig. S2A and S2B). Moreover, both protein and mRNA levels of C/EBPα and PPARγ were notably up-regulated in *Usp17*-knockdown cells (Fig. 1F and G; Fig. S2C and S2D). In contrast, overexpression of *USP17* showed the opposite effects (Fig. S2E–S2I). These results indicate that USP17 is a negative regulator of 3T3-L1 adipocyte differentiation. To gain deeper insights into the mechanism underlying USP17-mediated inhibition of adipocyte differentiation, we utilized a mutant form of USP17 (Fig. 1H; Fig. S2J). The mutant had its Cys 89 residue, which is critical for USP's catalytic activity, replaced with Ser, rendering the enzyme catalytically inactive.^3^ Staining results revealed that the mutant USP17 (C89S) reversed the inhibitory effects of wild-type USP17 on fat droplet formation (Fig. 1I; Fig. S2K). Moreover, the mutant restored the reduced protein levels of C/EBPα, PPARγ, and adiponectin observed in cells transfected with wild-type USP17 (Fig. 1J; Fig. S2L). Together, these findings suggest that the catalytic activity of USP17 is paramount for its suppression of adipogenesis.

Emerging evidence highlights the pivotal role of USP17's catalytic activity in regulating the activity of HDAC family members, including HDAC2.^3^ Given that HDAC1, a member of the HDAC2 subfamily, inhibits adipocyte differentiation by interacting with PPARγ and suppressing its transcriptional activity,^4^ we investigated whether USP17 influenced HDAC1 stability. Wild-type USP17, but not its catalytic mutant USP17 (C89S), significantly increased HDAC1 protein levels in a dose-dependent manner (Fig. S3A; Fig. 1K). Furthermore, USP17 overexpression markedly extended HDAC1's half-life in the presence of the protein synthesis inhibitor cycloheximide, whereas USP17 (C89S) had no effect (Fig. 1L), suggesting that USP17 enhances the steady-state levels of HDAC1. Next, we assessed the interaction between USP17 and HDAC1 via immunoprecipitation. Ectopically expressed HA-tagged USP17 and Myc-tagged HDAC1 co-immunoprecipitated, confirming their reciprocal interaction, whereas no signal was detected with control IgG (Fig. 1M). We then investigated whether USP17 regulated HDAC1 stability through deubiquitination. Overexpression of wild-type USP17 significantly reduced HDAC1 ubiquitination, whereas USP17 (C89S) was unable to do so. Notably, USP17 (C89S) still bound to HDAC1, indicating that Cys 89 is not required for their reciprocal interactions (Fig. 1N). Collectively, these results identified HDAC1 as a novel substrate of USP17.

Since PPARγ is a central regulator of adipogenesis, we examined whether USP17 influenced its transcriptional activity. When co-expressed with PPARγ, wild-type USP17 dramatically suppressed the activity of aP2- or PPRE-luciferase reporters, whereas USP17 (C89S) nearly abolished this suppression. These effects were further amplified by rosiglitazone, a well-known agonist of PPARγ (Fig. 1O; Fig. S3B). Furthermore, HDAC1 not only inhibited PPARγ transcriptional activity but also reduced the enhanced activity induced by USP17 (C89S) (Fig. 1P; Fig. S3C). Collectively, USP17 suppresses PPARγ transcriptional activity, partially via HDAC1. Adiponectin, a major adipokine mediating protection against obesity and cardiovascular disease, exhibits reciprocal regulation with PPARγ: PPARγ up-regulates adiponectin, while adiponectin enhances PPARγ activity through mechanisms including AMPK signaling and anti-inflammatory effects.^5^ Therefore, deciphering crosstalk between USP17 and the adiponectin–PPARγ axis represents a potential therapeutic target for obesity.

In conclusion, our research highlights the paramount role of USP17 as a negative regulator of 3T3-L1 adipocyte differentiation. By deubiquitinating and stabilizing HDAC1, USP17 suppresses the expression and activity of critical adipogenic regulators, including C/EBPα and PPARγ, thereby inhibiting lipid accumulation. The USP17–HDAC1 axis presents a promising therapeutic target for obesity, with the ability to modulate adipogenesis. Future research should focus on the therapeutic potential of USP17 inhibition in clinical settings to provide new options for treating obesity and associated metabolic disorders.

## CRediT authorship contribution statement

**Yuankuan Li:** Writing – original draft, Visualization, Methodology, Investigation, Formal analysis, Data curation. **Meiyu Piao:** Writing – review & editing, Visualization, Methodology, Investigation, Formal analysis, Data curation. **Yujian Jin:** Writing – review & editing, Methodology, Investigation, Formal analysis. **Sung Ho Lee:** Writing – review & editing, Visualization, Supervision, Project administration, Methodology, Conceptualization. **Kwang Youl Lee:** Writing – review & editing, Supervision, Resources, Project administration, Methodology, Funding acquisition, Conceptualization.

## Data availability

The data presented in this study are available on request from the corresponding authors.

## Funding

This research was supported by the National Research Foundation (NRF) of Korea under the Ministry of Science and Technology (MSIT) (No. 2019R1A5A2027521).

## Conflict of interests

The authors declared no conflict of interests.
