# Supplementary material for: Deubiquitinase USP17 negatively regulates 3T3-L1 adipocyte differentiation via HDAC1
Source: Genes Dis. 2025 Nov 12;13(3):101930. doi: 10.1016/j.gendis.2025.101930 (PMC12824903; doi:10.1016/j.gendis.2025.101930)
Supplement: Multimedia component 1 [file mmc1.docx]

**Materials and Methods**

**Cell culture**

Human embryonic kidney (HEK) 293T cells and 3T3-L1 murine preadipocytes were obtained from the American Type Culture Collection (Manassas, VA, USA). These cells were cultured in Dulbecco’s Modified Eagle Medium (DMEM, #12100046, Gibco™, Carlsbad, CA, USA) supplemented with 10% fetal bovine serum (FBS, 16000-044, Gibco™) and 1% antibiotic–antimycotic (#15240062; Gibco™). The cells were maintained in an incubator at 37 °C with saturated humidity and 5% CO_2_.

**3T3-L1 adipocyte differentiation**

Two days after reaching confluence, growth-arrested 3T3-L1 preadipocytes were treated with a differentiation cocktail (MDI) comprising 5 μg/mL insulin (Sigma-Aldrich, St. Louis, MO, USA), 1 μM Dex (Sigma-Aldrich), and 0.5 mM 3-isobutyl-1-methylxanthine (IBMX, Sigma-Aldrich) in the culture medium (10% FBS-DMEM) to initiate adipocyte differentiation (Day 0). To enhance differentiation efficacy, the transfected cells were also treated with 0.25 μM rosiglitazone in addition to MDI. Two days after treatment with MDI (Day 2), the cells were treated with 5 μg/mL insulin in the culture medium for an additional two days. Thereafter, the cells were maintained in the culture medium, with medium changes occurring every two days. On Day 8, 3T3-L1 preadipocytes differentiated into mature adipocytes, which were then used for experiments.

**Plasmids and transfection**

HDAC1, PPARγ, ubiquitin (Ub), and USP17 plasmids were cloned into a CMV promoter-derived mammalian expression vector (pCS4+), each tagged with HA, Myc, or Flag. The plasmids for Myc-tagged wild-type USP17 and its catalytic mutant USP17 (C89S) were kindly provided by Professor Kwang-Hyun Baek (CHA University, Gyeonggi-do, South Korea). Luciferase reporter plasmids aP2-luciferase (aP2-Luc), PPRE-luciferase (PPRE-Luc) and β-galactosidase (β-gal) were cloned into the pGL3 Basic and pCMV vectors, respectively. The luciferase reporter plasmid PPRE-Luc features the consensus PPAR responsive element (PPRE), whereas the luciferase reporter plasmid aP2-Luc comprises the aP2 promoter region, which bears the PPREs. The small hairpin RNAs (shRNAs) for gene knockdown were constructed using the pSUPER.retro.puro vector (Oligoengine, Seattle, WA, USA) following the manufacturer’s protocol. The sequences of the described shRNAs were as follows: shNr3c1, 5’-GAGGTGTTATATGCAGGATAT-3’; shUsp17, 5’-GGCTGTAAGATGTGTGCTA-3’. Polyethyleneimine (PEI) (Polysciences, Inc., Warrington, PA, USA) was used as the transfection agent for the aforementioned plasmids for both 3T3-L1 and HEK 293T cells.

**Oil Red O staining**

The differentiated 3T3-L1 cells were washed twice with phosphate-buffered saline (PBS) and fixed using 10% formalin for 10 min at room temperature. The fixed cells were rinsed briefly with 60% isopropanol for 1 min. They were then stained with 0.5% Oil Red O (O0625; Sigma-Aldirch) for 20 min. Following staining, the excess dye was removed by rinsing the cells with 60% isopropanol for 30 s, followed by several PBS washes. The stained cells were imaged using an inverted microscope (Niko Company, Japan) equipped with the NIS-Elements software. To quantify lipid content, the Oil Red O stain was dissolved in 100% isopropanol, and the absorbance at 510 nm was measured using a microplate reader (Epoch, Bio-Tek Instruments, Winooski, VT, USA).

**Triglyceride (TG) colorimetric assay**

Intracellular TG content in mature 3T3-L1 adipocytes was visualized using the TG Colorimetric Assay Kit (E-BC-K261-M; Elabscience Biotechnology Inc., Houston, TX, USA) following the manufacturer’s protocols. Briefly, 100% isopropanol was added to fully differentiated 3T3-L1 cells, followed by centrifugation at 1,000 × g for 10 min. For the assay, 2.5 µL of the supernatant was mixed with 250 µL of the reagent in a 96-well plate and incubated at 37 °C for 10 min. The absorbance at 510 nm was then measured using a microplate reader for TG quantification.

**Immunoblotting and** **immunoprecipitation**

Total cell lysates were prepared using an ice-cold lysis buffer (0.25% sodium deoxycholate, 1% Nonidet P-40, 10% glycerol, 1 mM Na_3_VO_4_, 1 mM EDTA, 25 mM HEPES at pH 7.5, and 150 mM NaCl) supplemented with protease and phosphatase inhibitors (250 mM PMSF, 25 mM NaF, 10 mg/mL leupeptin, and 10 mg/mL aprotinin). The mixtures were centrifuged at 13,000 × g for 15 min at 4 °C. Equal amounts of proteins were separated by sodium dodecyl sulfate-polyacrylamide gel electrophoresis (SDS-PAGE) and transferred to polyvinylidene difluoride membranes (Immobilon-P, Millipore, Burlington, MA, USA). The membranes were blocked with 5% skim milk and incubated overnight at 4 °C with indicated primary antibodies (**Table S1**). Next, the membranes were incubated for 1 h at room temperature with horseradish peroxidase-conjugated secondary antibodies. Protein bands were visualized using the Amersham^TM^ ImageQuant^TM^ 800 system (GE Healthcare Life Sciences, Marlborough, MA, USA), and band intensities were quantified using the ImageJ software (Version 1.42q, National Institutes of Health, Bethesda, MD, USA). For immunoprecipitation, equal amounts of proteins were incubated overnight at 4 °C with anti-HA or anti-Myc antibodies, or IgG as the control, under gentle shaking. The antibody complexes were conjugated with the Protein A Sepharose CL-4B (#17096303; GE Healthcare Life Sciences) for 4 h at 4 °C. The beads were washed thrice with the lysis buffer, resuspended in 5x loading buffer (100 µL bromophenol blue, 5 mL 1 M Tris at pH 7.5, 25 mL 10% SDS, 20 mL glycerol, and 50 mL distilled water), and boiled at 100 °C for 5 min. The samples were then analyzed by immunoblotting.

**Reverse transcription polymerase chain reaction (RT-PCR) and quantitative RT-PCR (RT-qPCR)**

Total RNA was extracted using the RNAiso Plus kit (TaKaRa, Tokyo, Japan), and RNA concentration was measured using the DS 11 Spectrophotometer Fluorometer (DeNovix Inc., Wilmington, DE, USA). Next, 1 µg of total RNA was reverse-transcribed into cDNA using the GoScript^TM^ Reverse Transcription System (Promega, Madison, WI, USA) following the manufacturer’s protocols. The RT-PCR cycle was as follows: initial denaturation at 94 °C for 3 min, further denaturation at 94 °C for 30 s, annealing at the primer-specific optimized temperature for 30 s, and extension at 72 °C for 20 s. This cycle was repeated 25–30 times, with the final extension conducted at 72 °C for 5 min. RT-qPCR was conducted using the CFX 96 real-time system (Bio-Rad, Hercules, CA, USA) using the TB Green® Premix Ex Taq™ (Tli RNaseH Plus) Kit (TaKaRa) following the manufacturer’s instructions. The primer sequences used for RT-PCR and RT-qPCR are listed in **Table S2**.

**Luciferase reporter assay**

HEK 293T cells were seeded into 24-well plates and transfected with luciferase reporter plasmids (aP2-Luc or PPRE-Luc), a β-galactosidase (β-gal) plasmid to normalize transfection efficiency, and various combinations of plasmids expressing PPARγ, HDAC1, and either wild-type USP17 or the mutant USP17 (C89S). After 24 h, the transfected cells were treated with 0.5 µM rosiglitazone or the corresponding volume of vehicle (DMSO). The luciferase activity in the transfected cells was measured 42 h post-transfection using a luciferase reporter assay kit (Promega).

**Deubiquitination assay**

HEK 293T cells were transfected with plasmids expressing Flag-tagged ubiquitin (Ub), HA-tagged HDAC1, and either wild-type USP17, the mutant USP17 (C89S), or the control vector. After 24 h, the cells were treated with 10 µM proteasome inhibitor MG132 for 8 h and harvested for immunoprecipitation. Equal amounts of lysates were incubated overnight at 4 °C with a Flag antibody, followed by incubation with Protein A Sepharose CL-4B for 4 h at 4 °C. The resulting immunoprecipitates were analyzed by SDS-PAGE.

**Protein half-life analysis**

HEK 293T cells were transfected with the indicated plasmids encoding HA-tagged HDAC1, Myc-tagged USP17, the mutant USP17 (C89S), and the control vector. At 24 h post-transfection, the cells were treated with the protein synthesis inhibitor cycloheximide (CHX, 50 μg/mL, C1988; Sigma-Aldrich) for varying durations (0, 6, 12, and 24 h). Cell lysates were collected, and HDAC1 protein half-life was determined by immunoblotting.

**Statistical analysis**

The results were analyzed using GraphPad Prism 8.0.2 (San Diego, CA, USA), with statistical significance assessed by one-way ANOVA followed by Dunnett's multiple comparisons test (for comparisons with the control group) or Tukey’s multiple comparison test (for comparisons among the test groups). A *p*-value < 0.05 was considered statistically significant. The results are presented as mean ± standard error of the mean (SEM). All experiments were conducted three times or more.

**Supplementary tables**

**Table S1.** List of primary antibodies used for immunoblotting.

| **Antibody** | **Order#** | **Company** | **Dilution** |
| --- | --- | --- | --- |
| adiponectin | C45B10 | Cell Signaling Technology, Danvers, MA, USA | 1:1000 |
| β-actin | sc-47778 | Santa Cruz Biotechnology, Santa Cruz, CA, USA | 1:1000 |
| C/EBPα | SC-61 | Santa Cruz Biotechnology, Santa Cruz, CA, USA | 1:1000 |
| Flag | F3165 | Sigma-Aldrich, St. Louis, MO, USA | 1:1000 |
| HA | 12CA5 | Roche Applied Science, Basel, Switzerland | 1:1000 |
| PPARγ | D69 | Cell Signaling Technology, Danvers, MA, USA | 1:1000 |
| Myc | 9E10 | Santa Cruz Biotechnology, Santa Cruz, CA, USA | 1:1000 |

**Table S2.** List of primers used for RT-PCR and RT-qPCR.

| **Gene** | **Forward Primer (5’–3’)** | **Reverse Primer (5’–3’)** |
| --- | --- | --- |
| *Actb* | GCAGGAGTACGATGAGTCCG | ACGCAGCTCAGTAACAGTCC |
| *Cebpa* | CAAGAACAGCAACGAGTACCG | GTCACTGGTCAACTCCAGCAC |
| *Cebpb* | ACCGGGTTTCGGGACTTGA | GTTGCGTCAGTCCCGTGTCCA |
| *Il6* | GCTACCAAACTGGATATAATCAGG | CCAGGTAGCTATGGTACTCCAGAA |
| *Nr3c1* | GGGCTATGAACTTCGCAGGCC | CTTCATCGGAGCACACCAGGC |
| *Pparg* | GCCCTTTGGTGACTTTATGGA | GCAGCAGGTTGCTTGGATG |
| *Usp17* | TTCACGTGGGGTCCACATTT | GGGTGTCCAGCTTGTTGTCTT |

**Supplemental Figures**

**
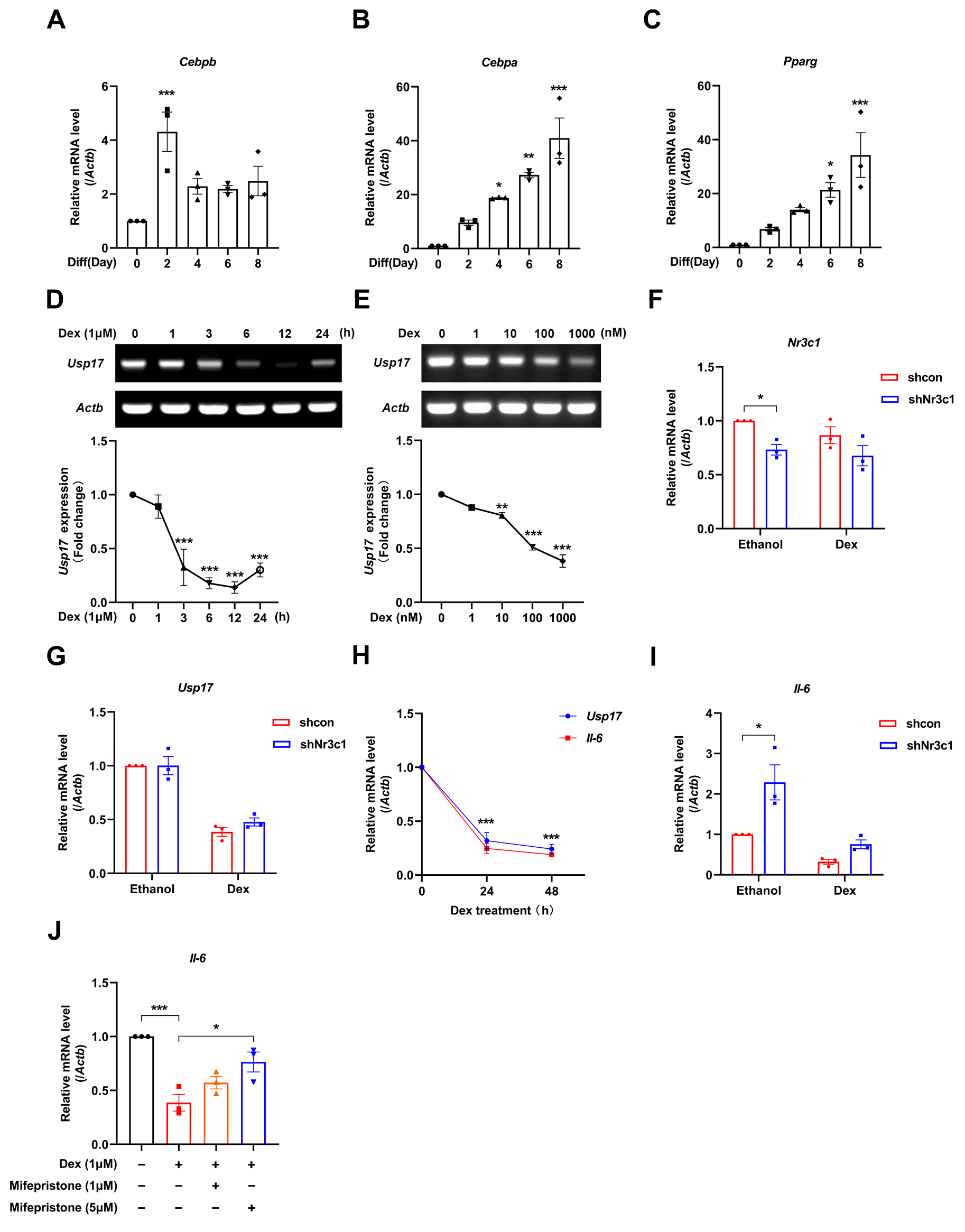
**

**Supplementary Figure 1.** USP17 expression dramatically declines during the differentiation of 3T3-L1 adipocytes, primarily driven by Dex via the GR/IL-6 axis. (**A–C**) The mRNA levels of *Cebpb*, *Cebpa*, and *Pparg* were measured in 3T3-L1 cells at different timepoints (Days 0, 2, 4, 6, and 8). **(D, E)** At 48 h post-confluence, 3T3-L1 cells were treated with 1 μM Dex for varying durations (0, 1, 3, 6, 12, assess 24 h) to assess its time-dependent effects on *Usp17* expression, or at different concentrations (0, 1, 10, 100, and 1000 nM) for 24 h to examine its dose-dependent effects. The mRNA level of *Usp17* was assessed using RT-PCR, and the band intensity was quantified using ImageJ, with *Actb* as the internal control. **(F, G and I)** The mRNA levels of *Nr3c1*, *Usp17*, and *Il-6* were measured in 3T3-L1 cells with or without *Nr3c1* knockdown, following the treatment of 1 μM Dex or ethanol for 24 h. **(H)** The mRNA levels of *Usp17* and *Il-6* were measured in 3T3-L1 cells treated with 1 μM Dex for indicated times (24 and 48 h). **(J)** The effect of mifepristone on Dex-induced decrease in *Il-6* mRNA levels. RT-qPCR was conducted to evaluate the mRNA levels, and *Actb* was used as the loading control. The results are presented as mean ± SEM. * *p* < 0.05, ** *p* < 0.01, *** *p* < 0.001. “Dex” indicates dexamethasone.


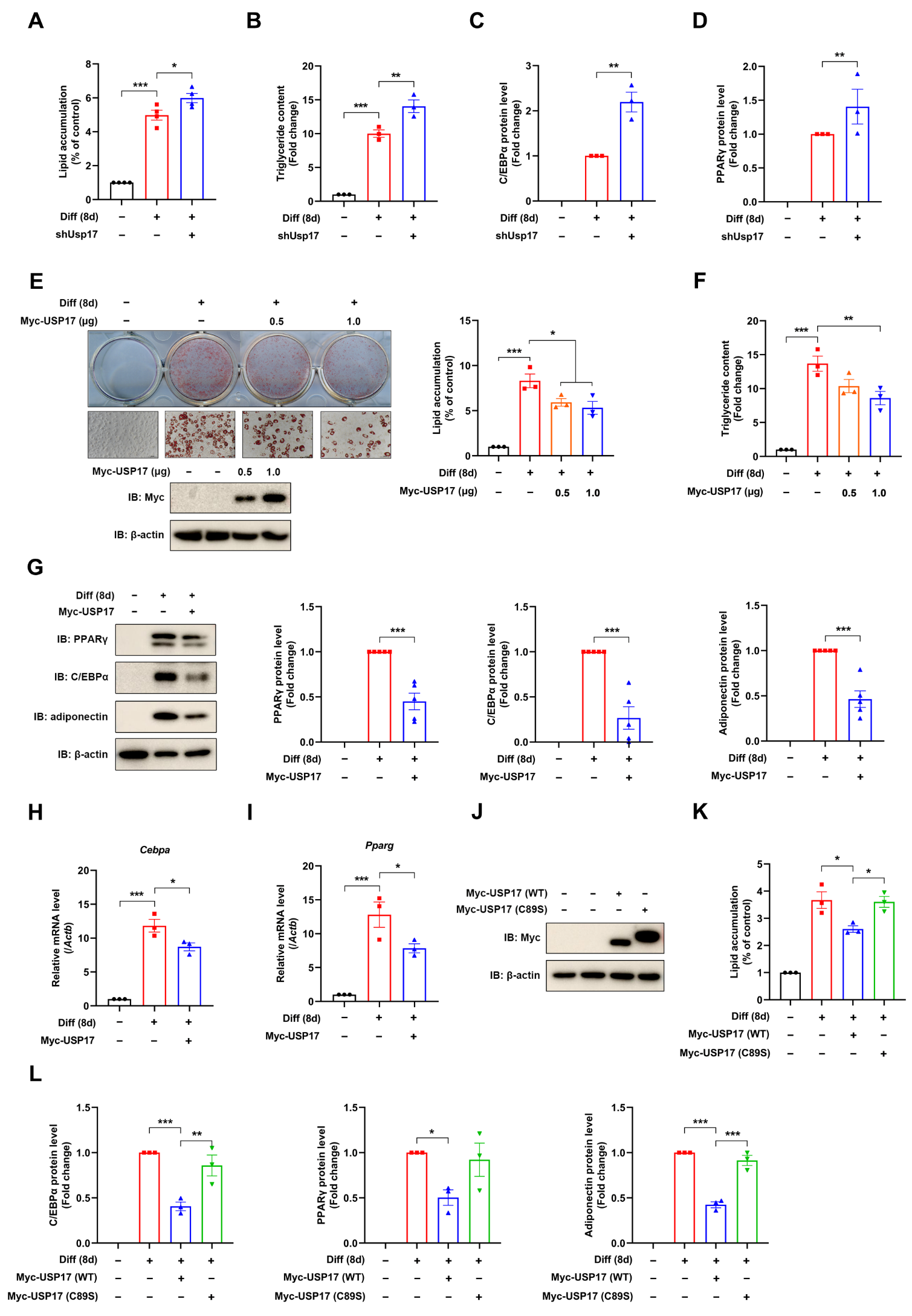


**Supplementary Figure 2.** USP17 negatively regulates 3T3-L1 adipocyte differentiation via its catalytic activity. After transfection, the two-day growth-arrested 3T3-L1 cells were primed for an eight-day adipogenic differentiation using the described differentiation cocktail. (**A, B**) The impact of *Usp17* knockdown on intracellular lipid accumulation and triglyceride levels in differentiated 3T3-L1 cells was assessed. **(C, D)** The intensities of bands corresponding to PPARγ and C/EBPα levels were normalized to the β-actin band intensity. (**E, F**) The effect of *USP17* overexpression on intracellular lipid accumulation and triglyceride levels in differentiated 3T3-L1 cells was assessed. Representative images of differentiated 3T3-L1 cells, with or without *USP17* overexpression, at 200x magnification. The scale bar represents 100 μm. The successful transfection of USP17 in 3T3-L1 cells was confirmed by immunoblotting (IB). (**G**) The protein levels of PPARγ, C/EBPα, and adiponectin were measured by IB and quantified relative to β-actin. **(H, I)** The effect of *USP17* overexpression on the mRNA levels of *Pparg* and *Cebpa* in differentiated 3T3-L1 cells was assessed using RT-qPCR. **(J)** IB was performed to confirm successful transfection of wild-type USP17 (WT) and mutant USP17 (C89S) in 3T3-L1 cells. **(K)** The effect of USP17's catalytic activity on intracellular lipid accumulation was quantified and expressed as a percentage relative to undifferentiated cells. **(L)** The protein levels of PPARγ, C/EBPα, and adiponectin were quantified relative to β-actin. The results are presented as mean ± SEM. * *p* < 0.05, ** *p* < 0.01, *** *p* < 0.001.


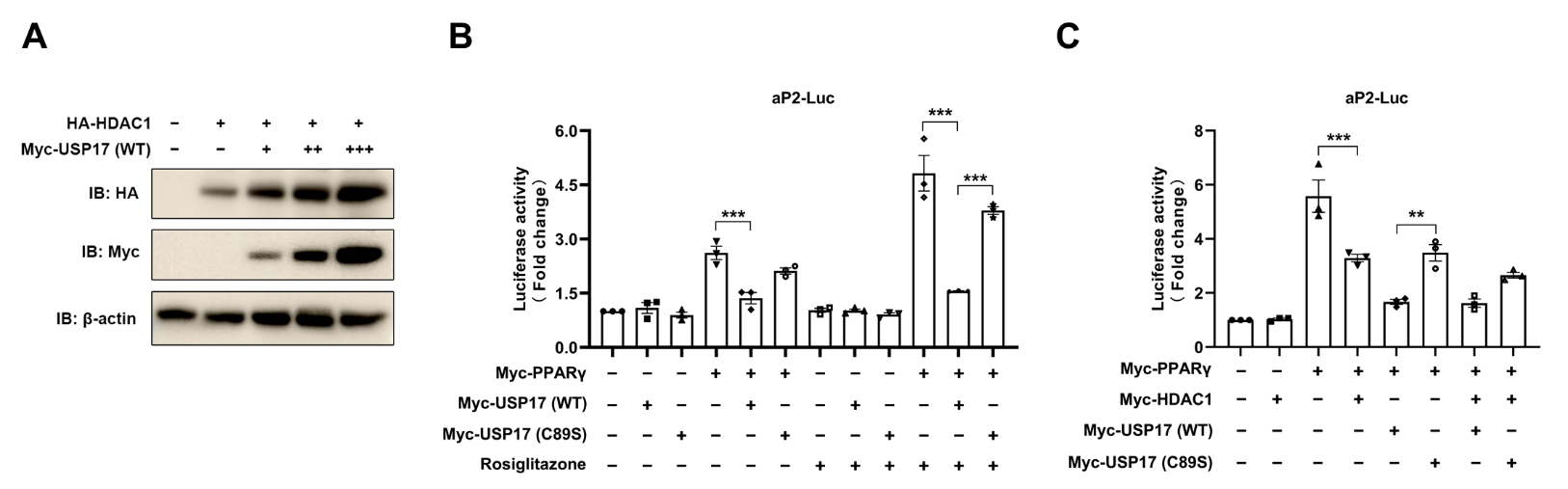


**Supplementary Figure 3.** USP17-mediated stabilization of HDAC1 partially suppresses PPARγ transcriptional activity. **(A)** HA-HDAC1 (0.5 μg) and varying amounts of Myc-USP17 (0.25, 0.5, and 1.0 μg) were co-transfected into HEK293T cells. The protein level of HDAC1 was measured by immunoblotting (IB) and normalized to that of β-actin. **(B)** To investigate the effects of wild-type USP17 and the catalytically inactive mutant USP17 (C89S) on PPARγ transcriptional activity, the indicated plasmids Myc-PPARγ and either Myc-USP17 (WT) or Myc-USP17 (C89S) were co-transfected into HEK293T cells along with the luciferase reporter plasmids (aP2-Luc) containing the aP2 promoter region with PPREs. At 24 hours post-transfection, the cells were treated with DMSO or 0.5 μM rosiglitazone, and luciferase activity was measured at 42 hours post-transfection. **(C)** HDAC1 diminished the positive effect of USP17 (C89S) on the transcriptional activities of the luciferase reporter plasmids (aP2-Luc). Luciferase activity is represented as fold changes in comparison to the control group. The results are shown as mean ± SEM. ** *p* < 0.01, *** *p* < 0.001.
