# Supplementary material for: Deubiquitinase USP17 negatively regulates 3T3-L1 adipocyte differentiation via HDAC1
Source: Genes Dis. 2025 Nov 12;13(3):101930. doi: 10.1016/j.gendis.2025.101930 (PMC12824903; doi:10.1016/j.gendis.2025.101930)

Figure 1F—The effect of *Usp17* knockdown on 3T3-L1 adipocyte differentiation

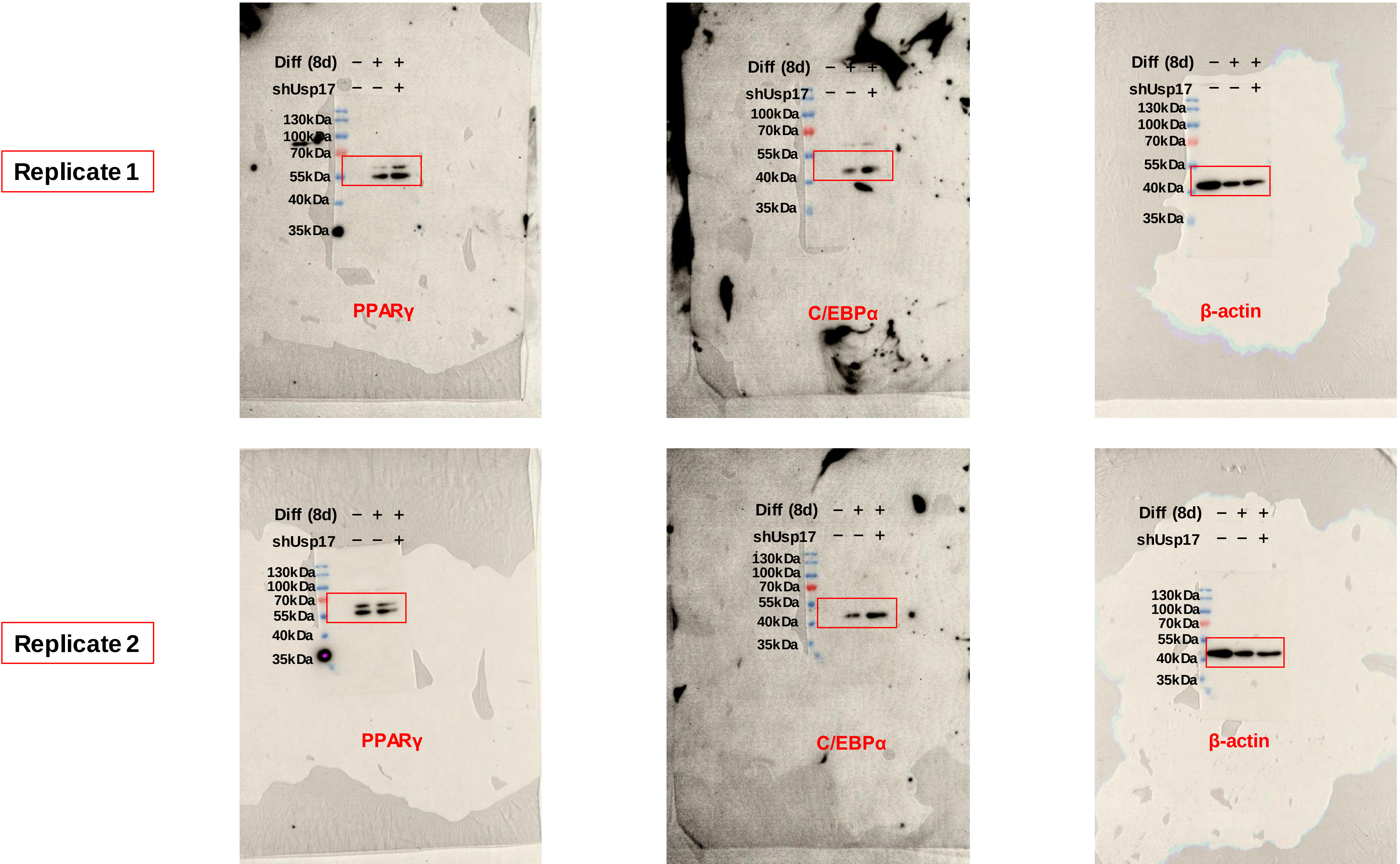

Figure 1F—The effect of *Usp17* knockdown on 3T3-L1 adipocyte differentiation

Replicate 3

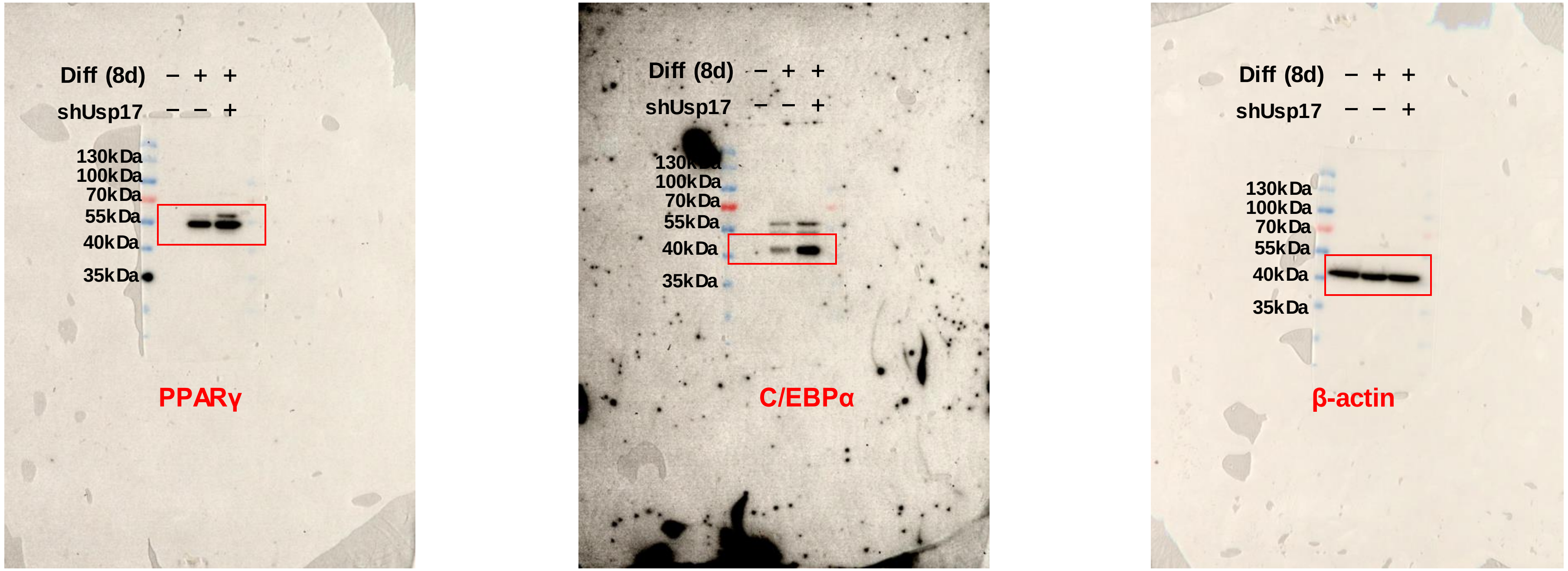

Figure 1J—The effect of catalytically inactive USP17 (C89S) on 3T3-L1 adipocyte differentiation

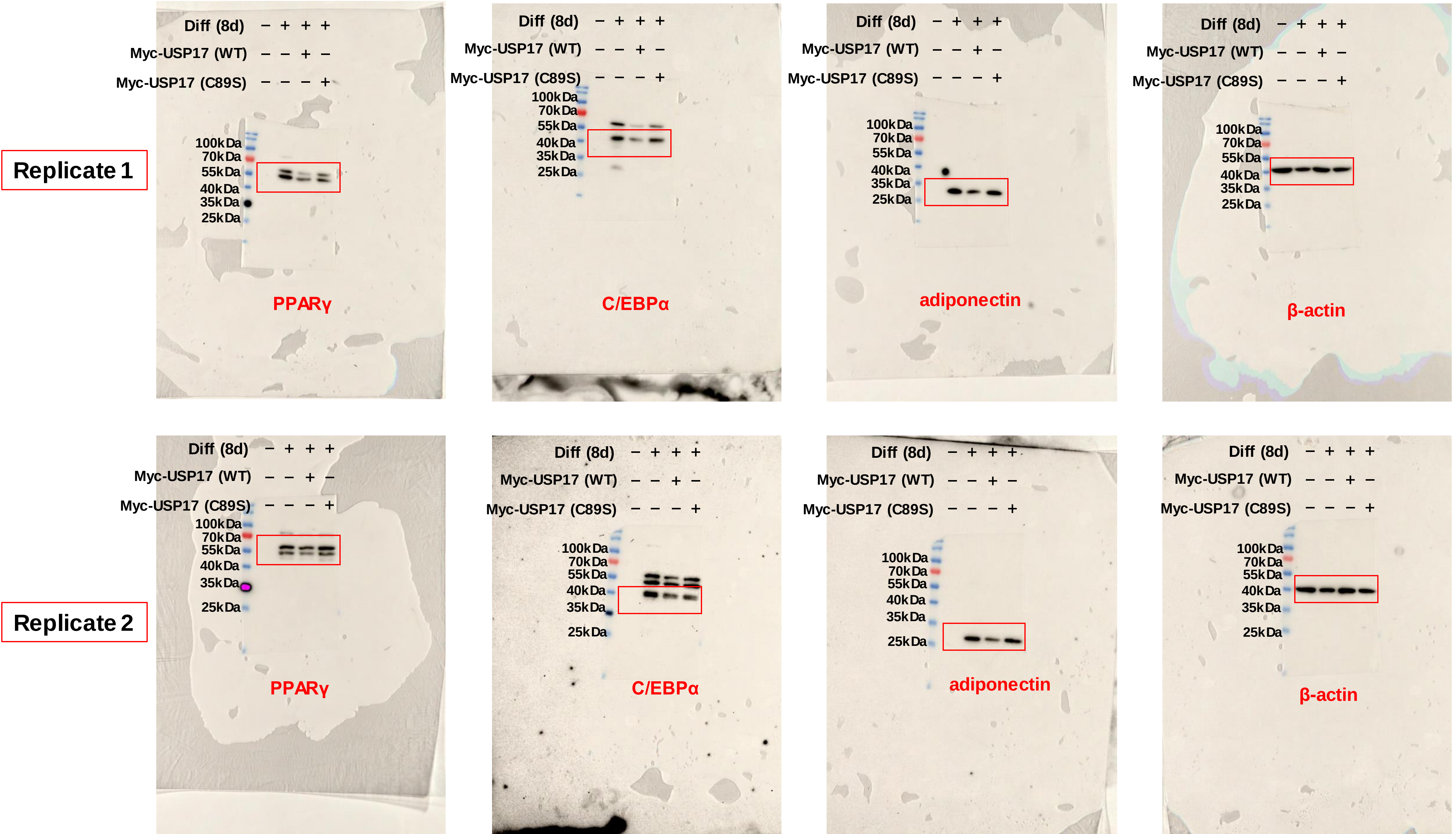

Figure 1J—The effect of catalytically inactive USP17 (C89S) on 3T3-L1 adipocyte differentiation

Replicate 3

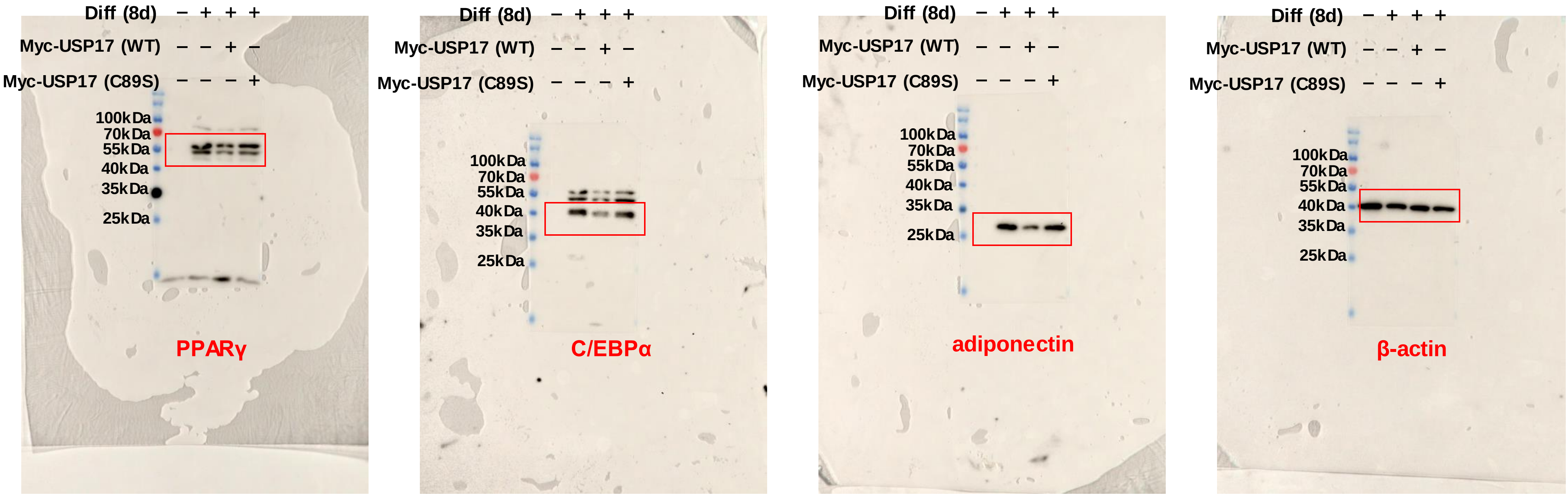

Figure 1K—The effect of catalytically inactive USP17 (C89S) on HDAC1 stability

Replicate 1

Replicate 2

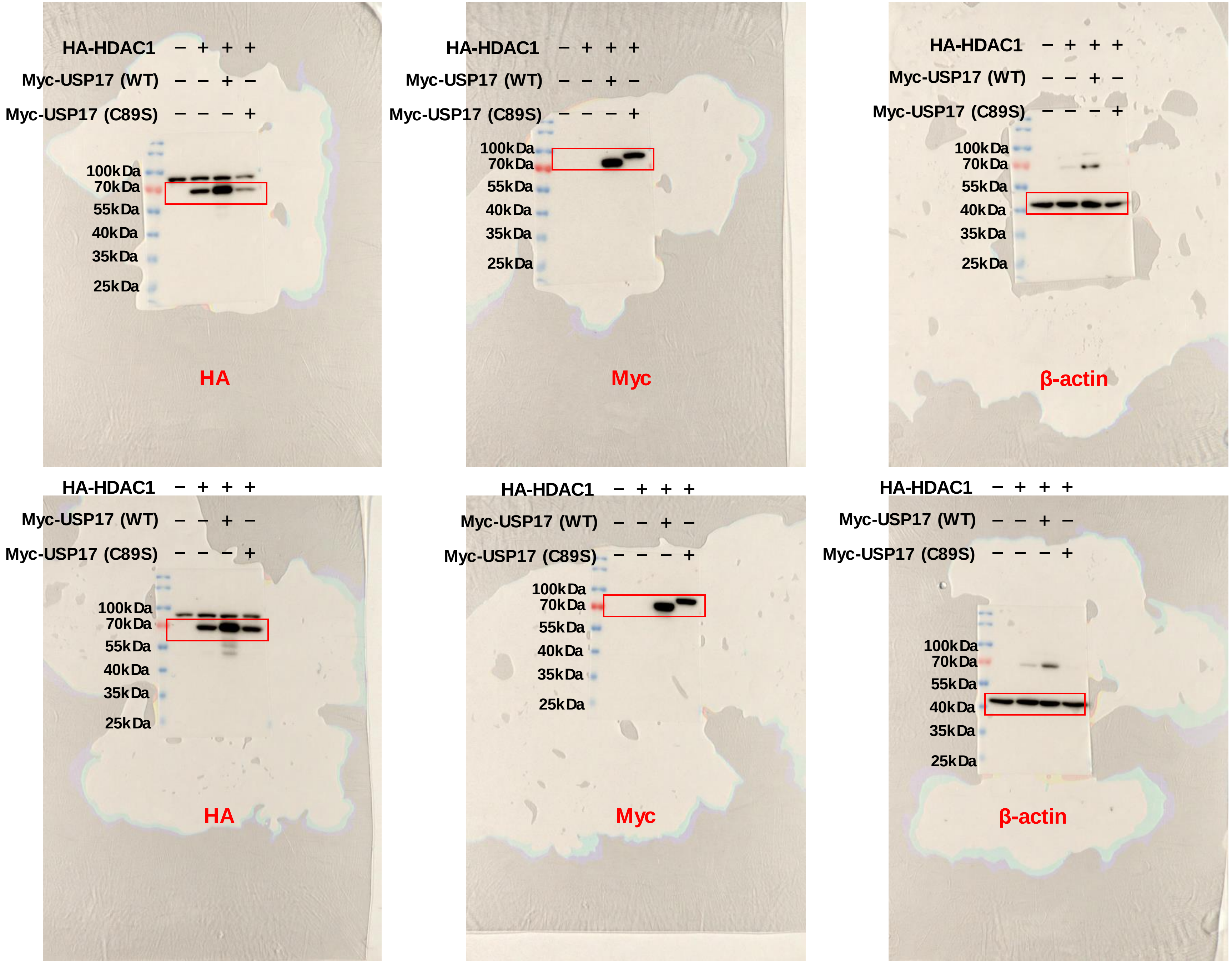

Figure 1K—The effect of catalytically inactive USP17 (C89S) on HDAC1 stability

Replicate 3

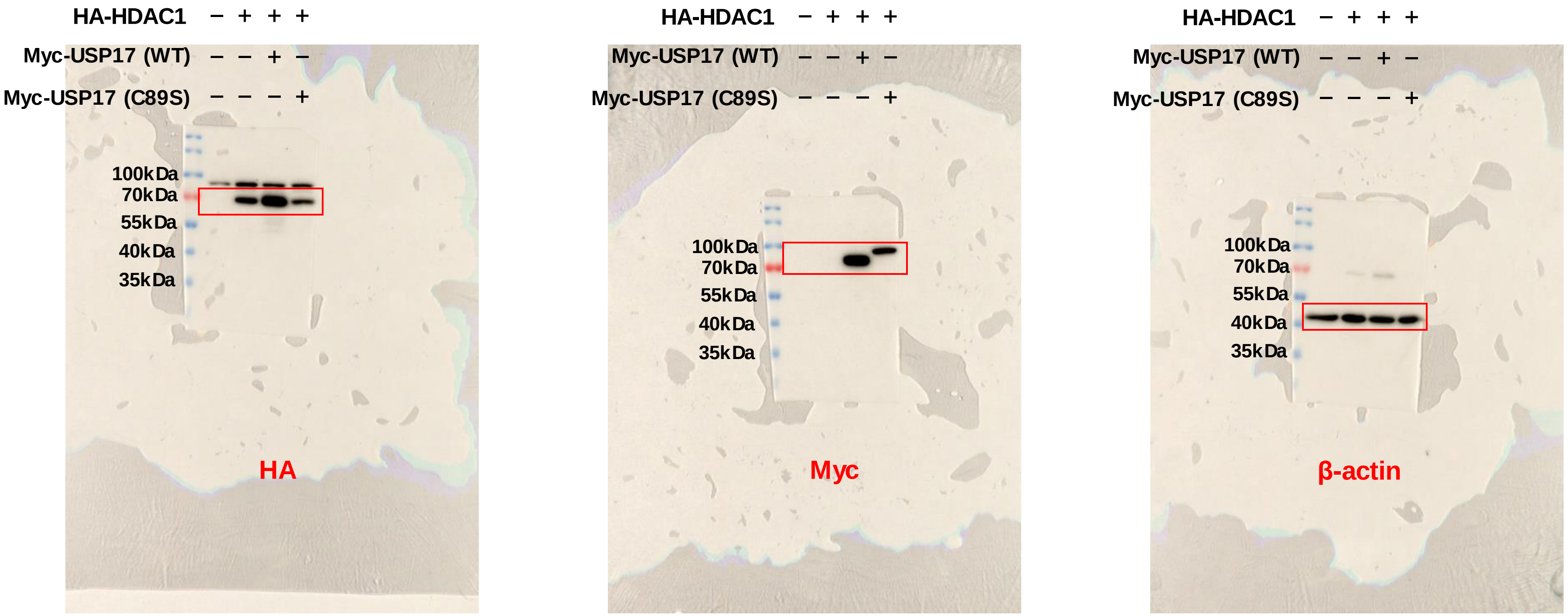

Figure 1L—The effect of USP17 (WT) and USP17 (C89S) on HDAC1 half-life

Replicate 1

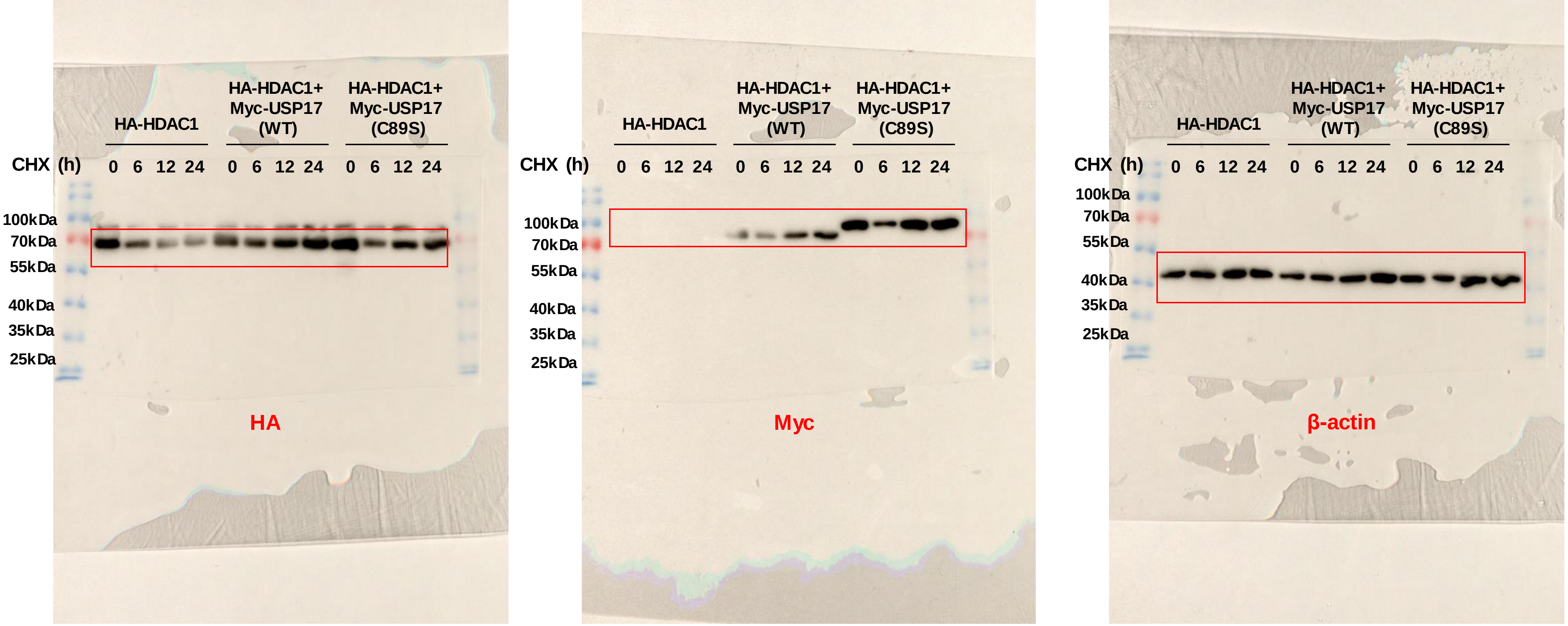

Figure 1L—The effect of USP17 (WT) and USP17 (C89S) on HDAC1 half-life

Replicate 2

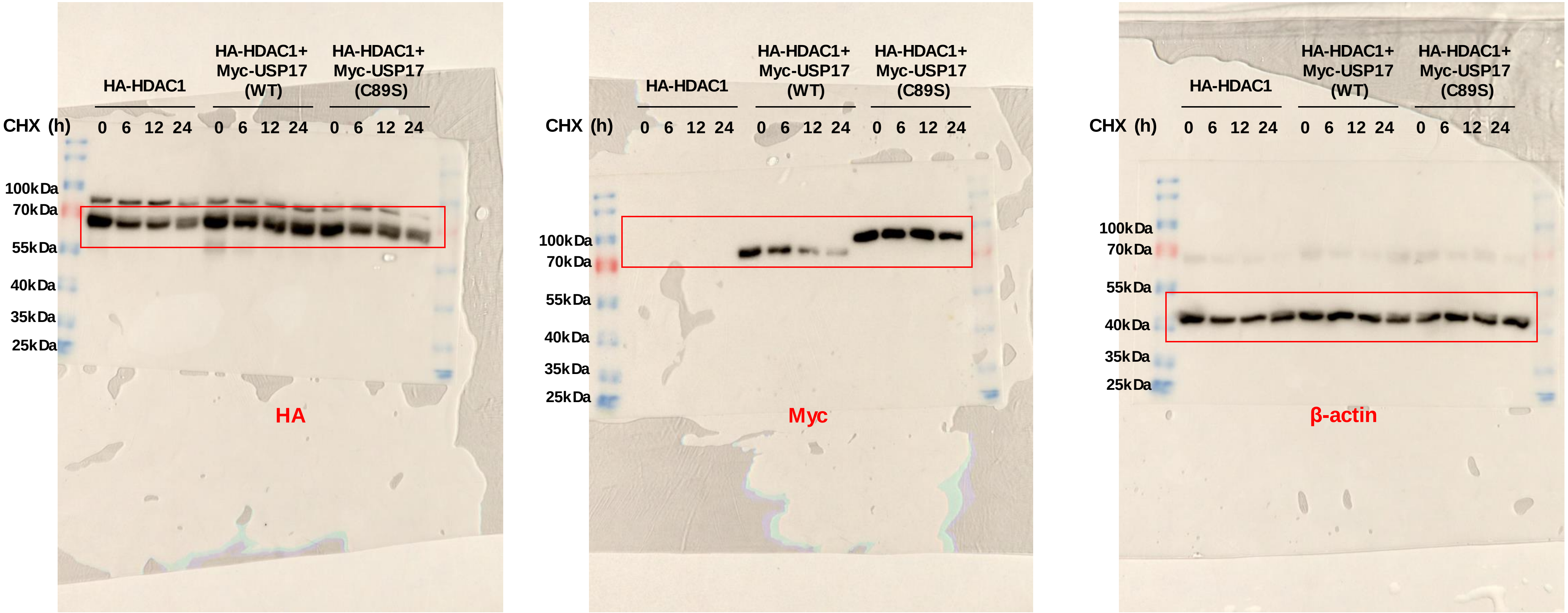

Figure 1L—The effect of USP17 (WT) and USP17 (C89S) on HDAC1 half-life

Replicate 3

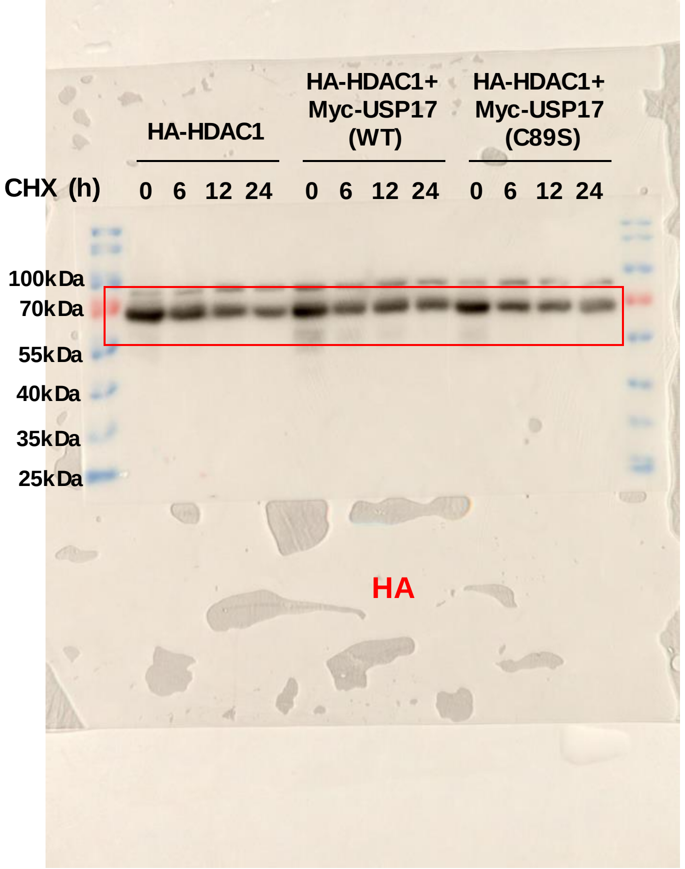

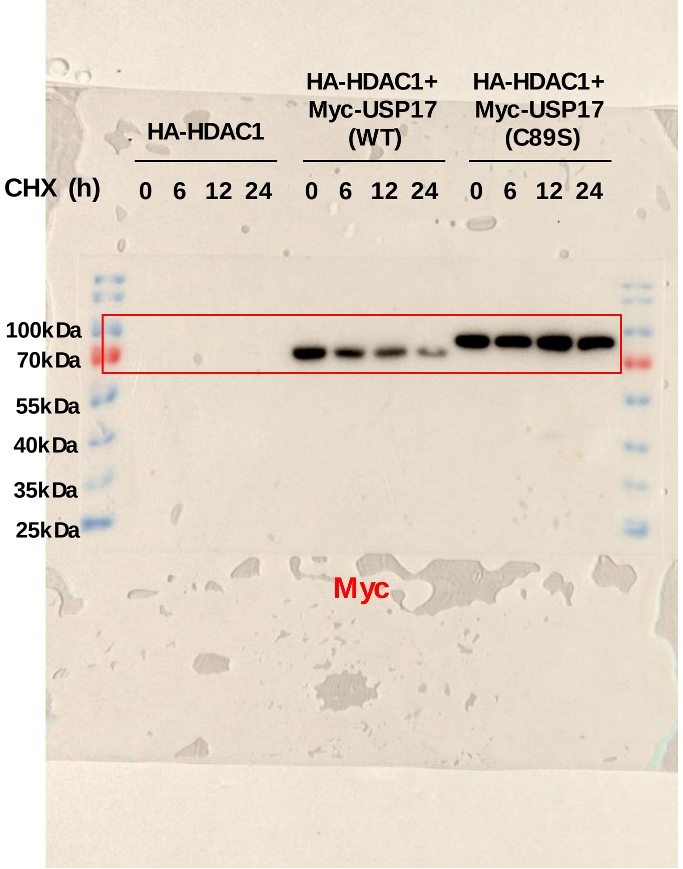

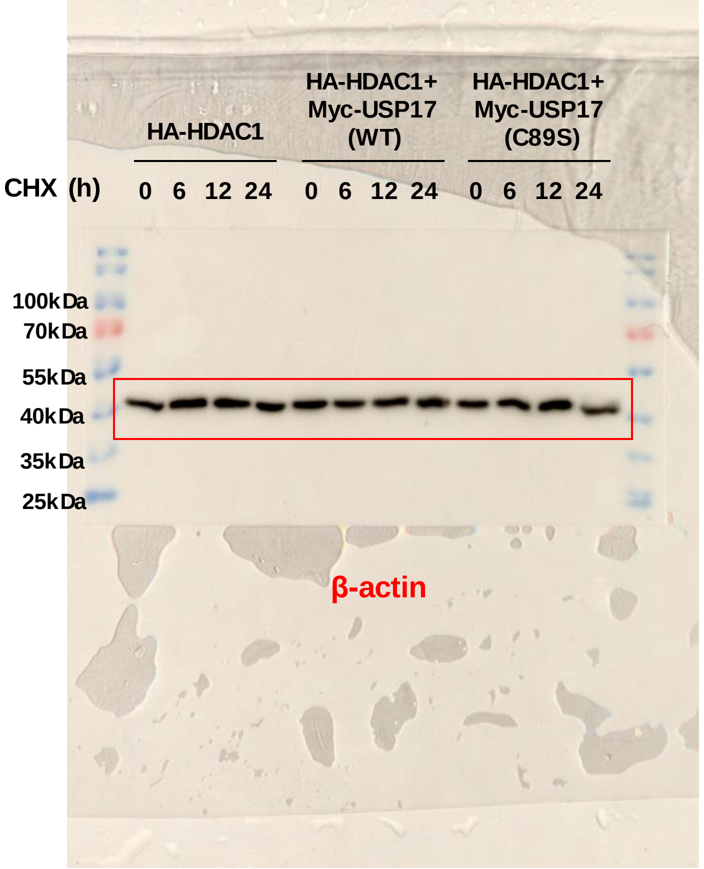

Figure 1L—The effect of USP17 (WT) and USP17 (C89S) on HDAC1 half-life

Replicate 4

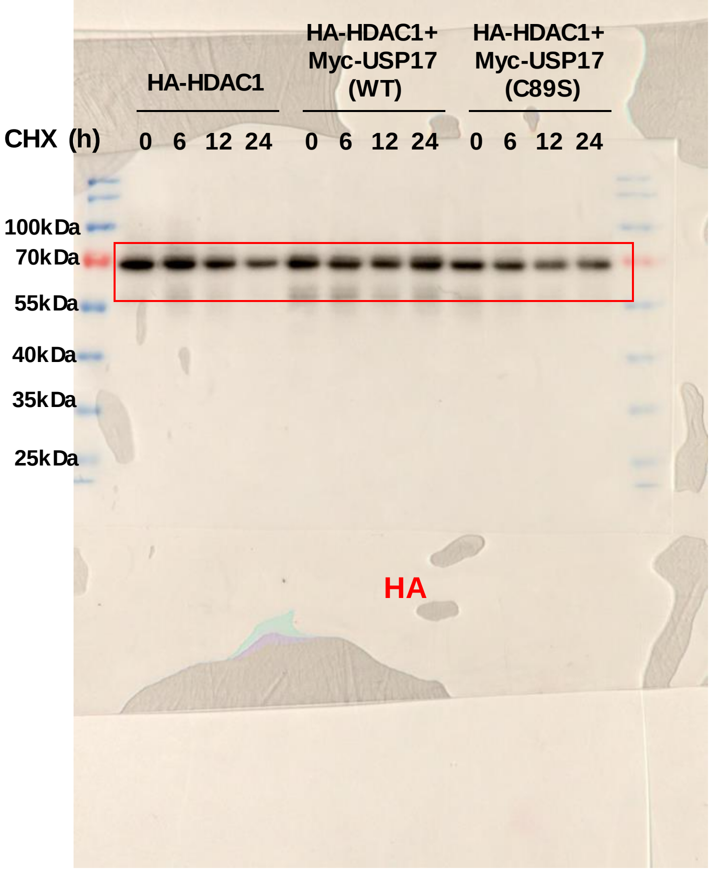

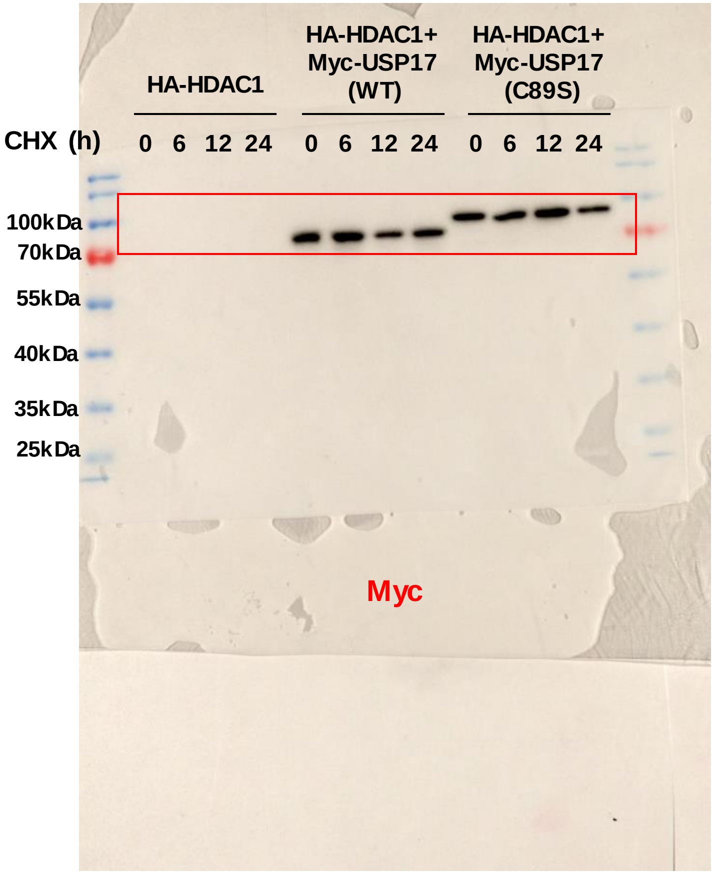

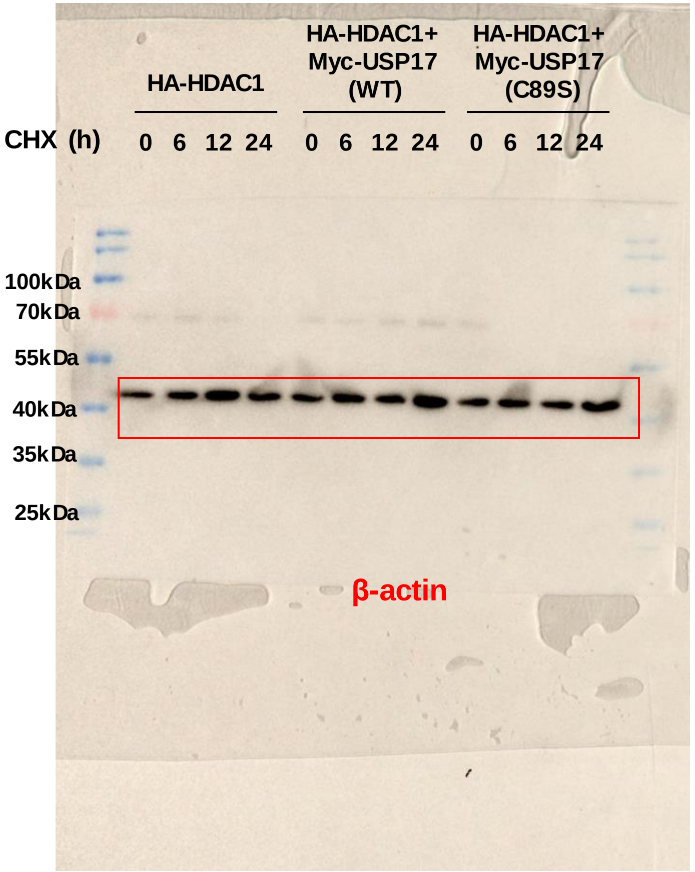

Figure 1M—HDAC1 binds with USP17

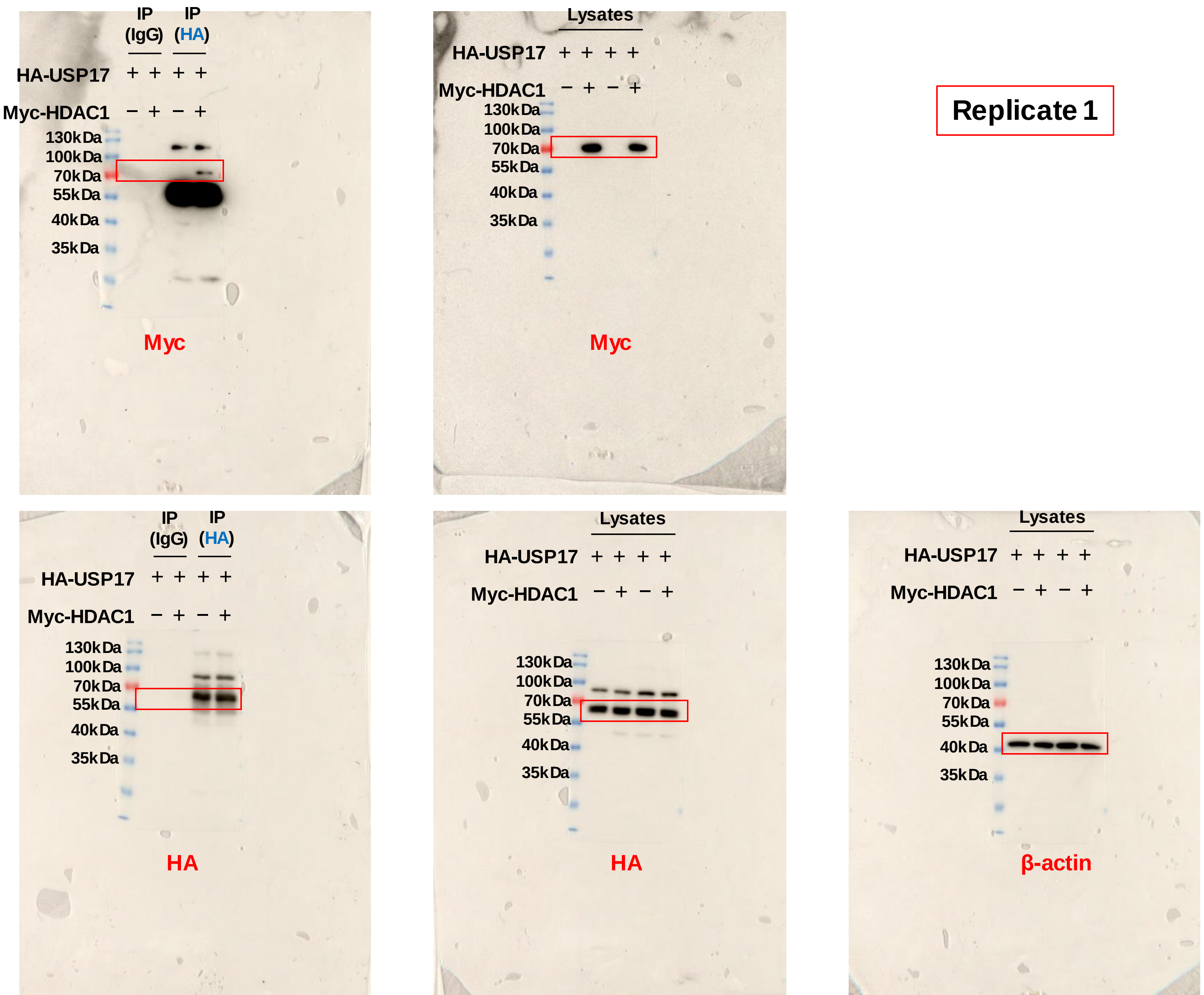

Figure 1M—HDAC1 binds with USP17

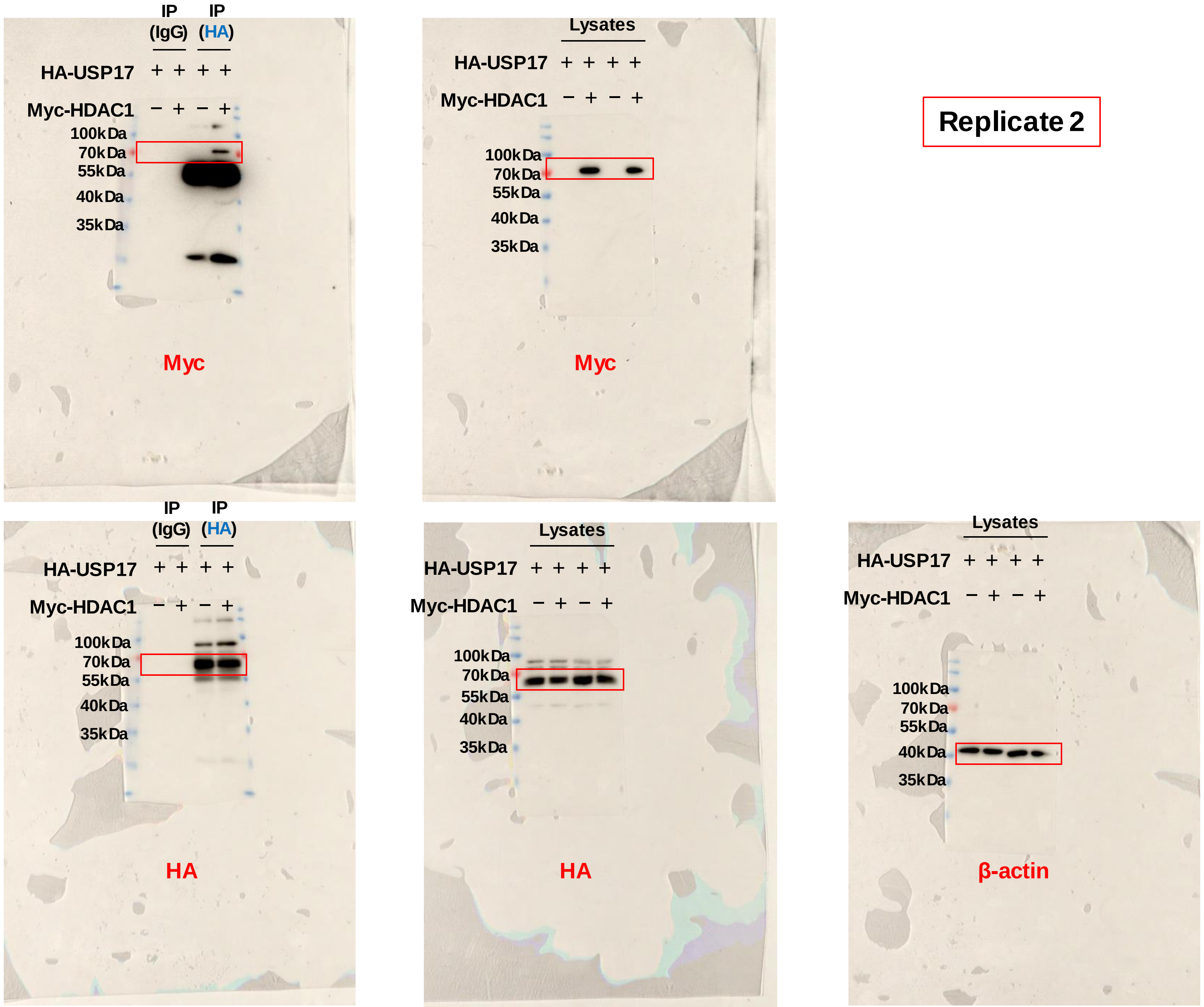

Figure 1M—HDAC1 binds with USP17

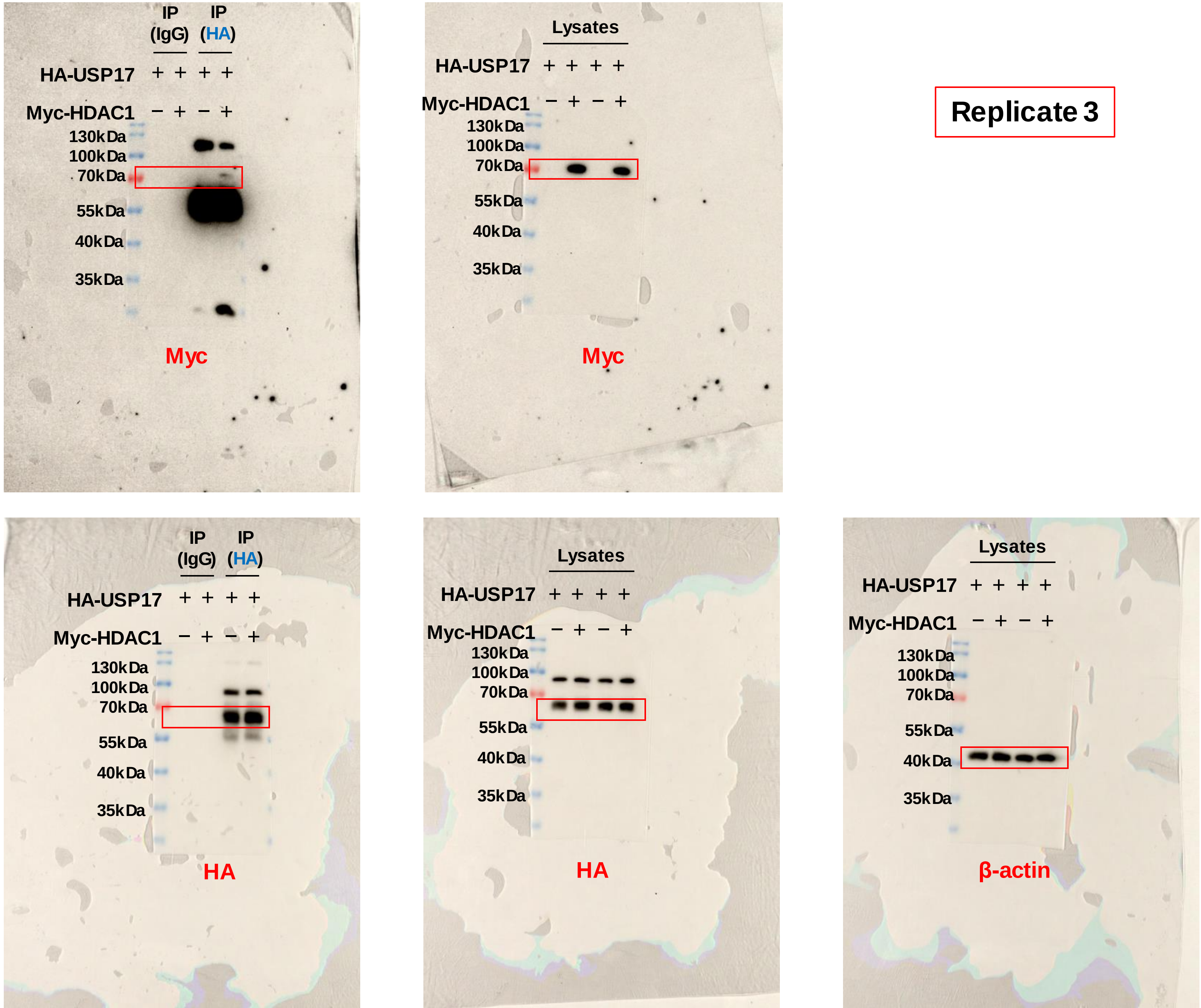

Figure 1M—USP17 binds with HDAC1

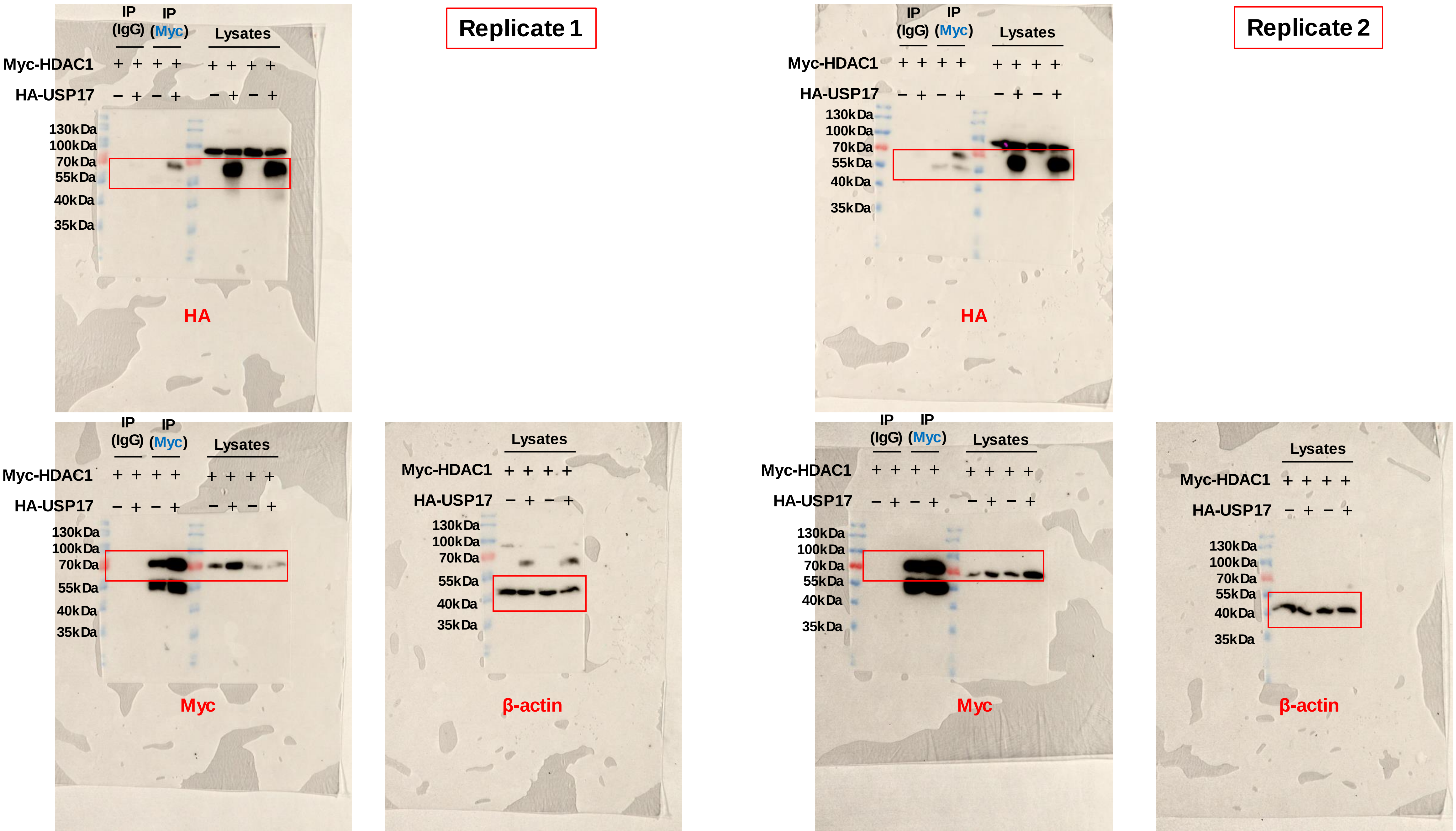

Figure 1M—USP17 binds with HDAC1

Replicate 3

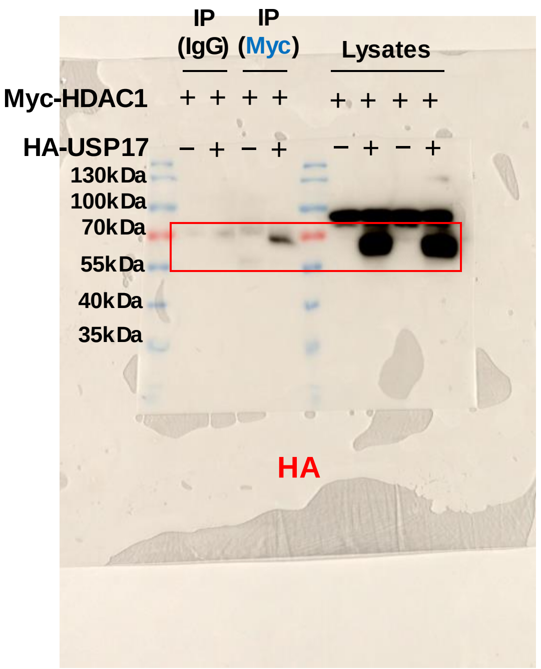

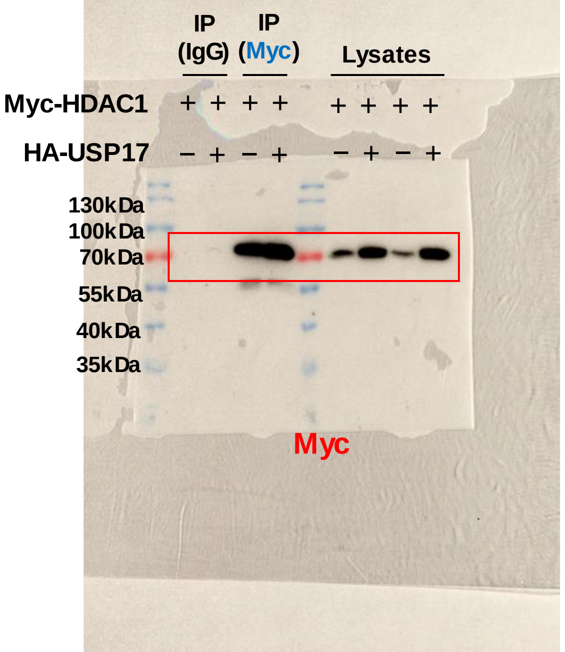

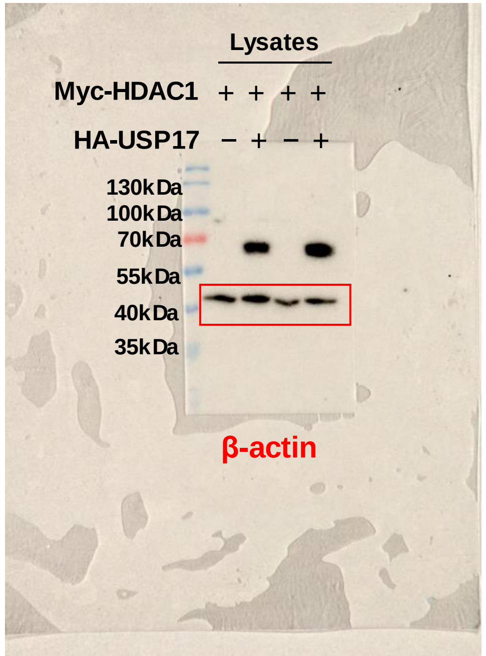

Figure 1N—USP17 deubiquitinates HDAC1

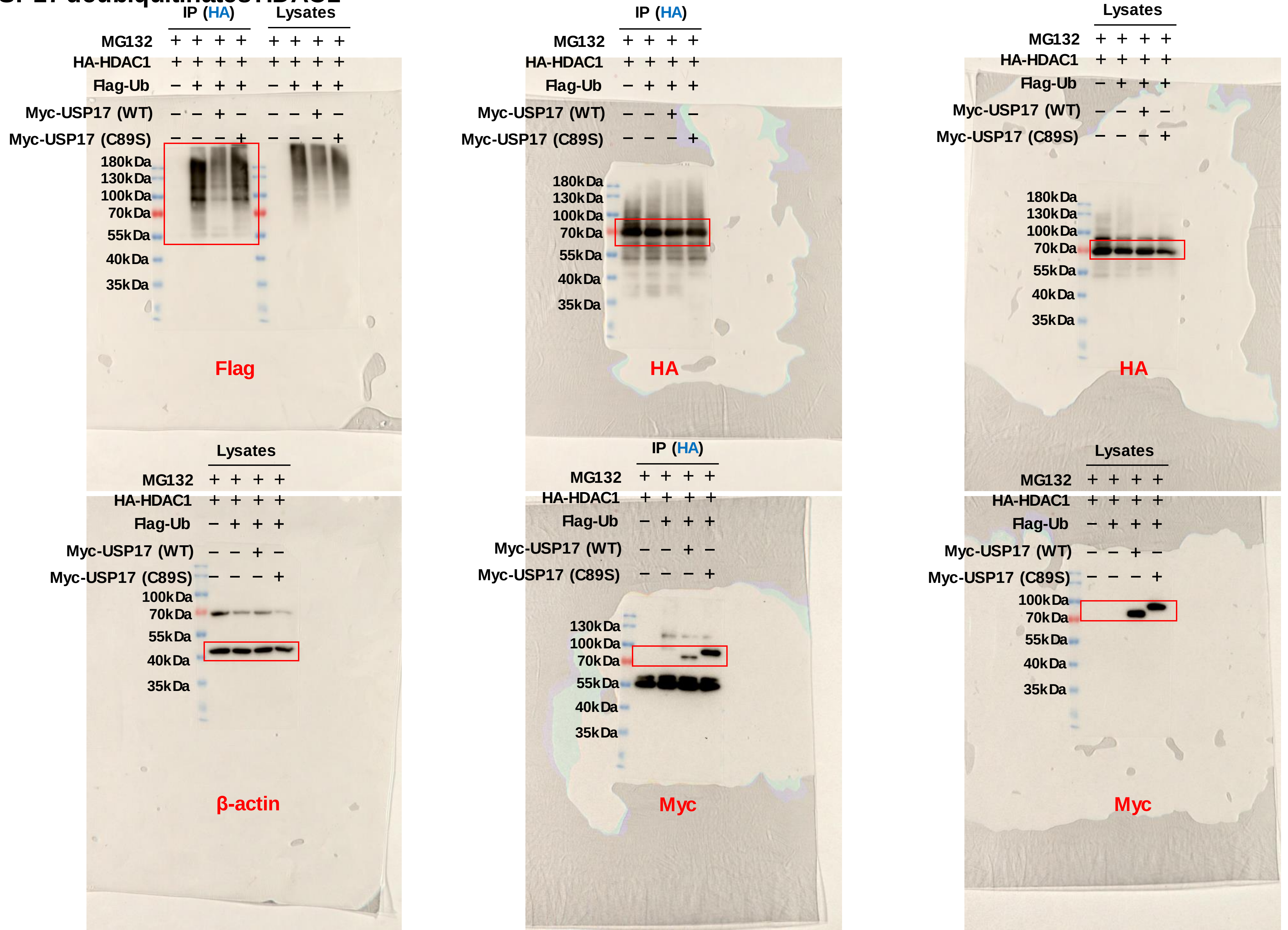

Replicate 1

Figure 1N—USP17 deubiquitinates HDAC1

Replicate 2

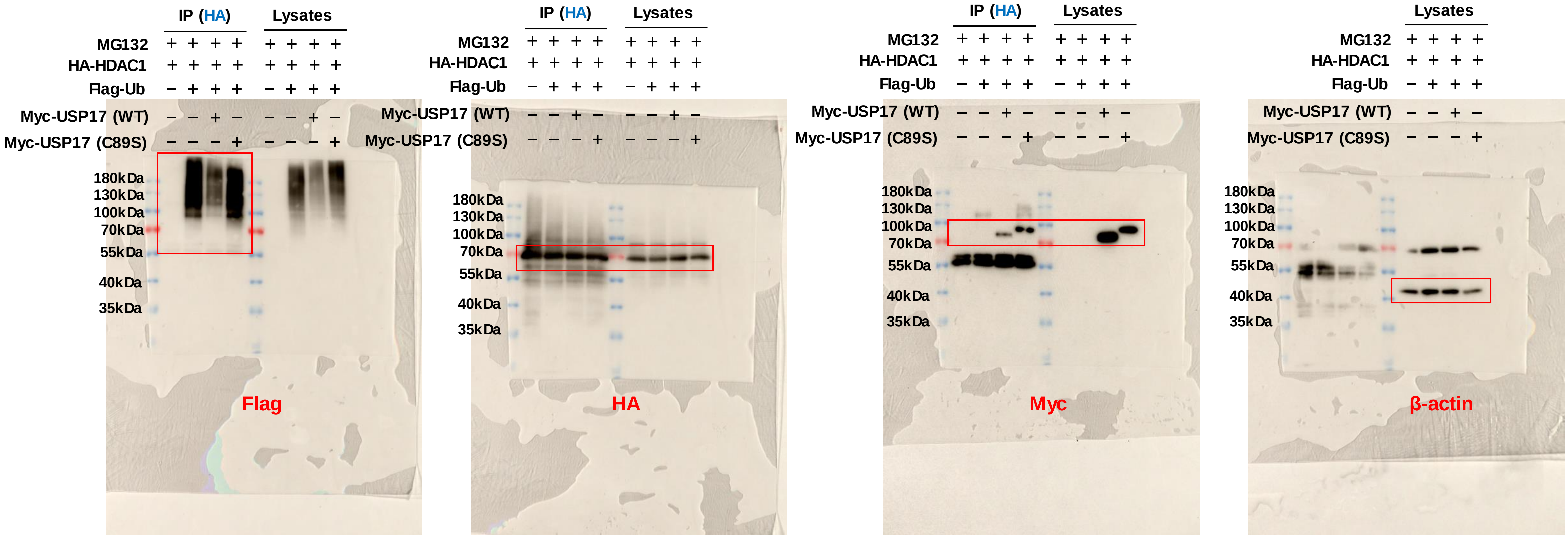

Figure 1N—USP17 deubiquitinates HDAC1

Replicate 3

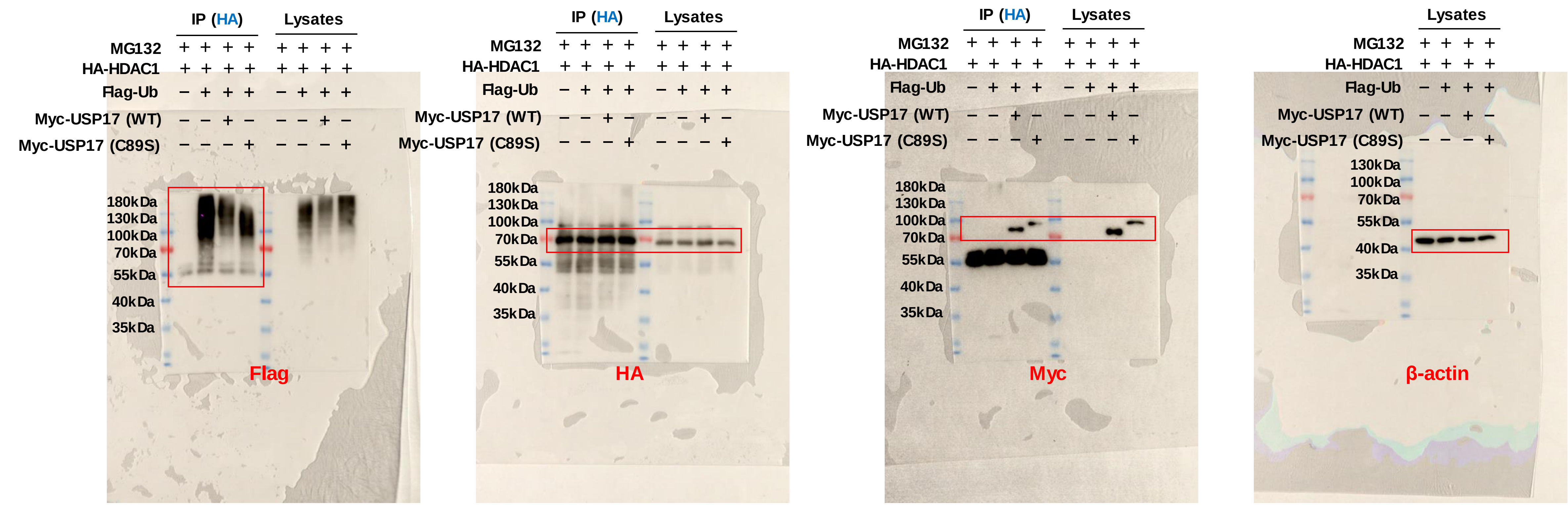

Figure 1N—USP17 deubiquitinates HDAC1

Replicate 4

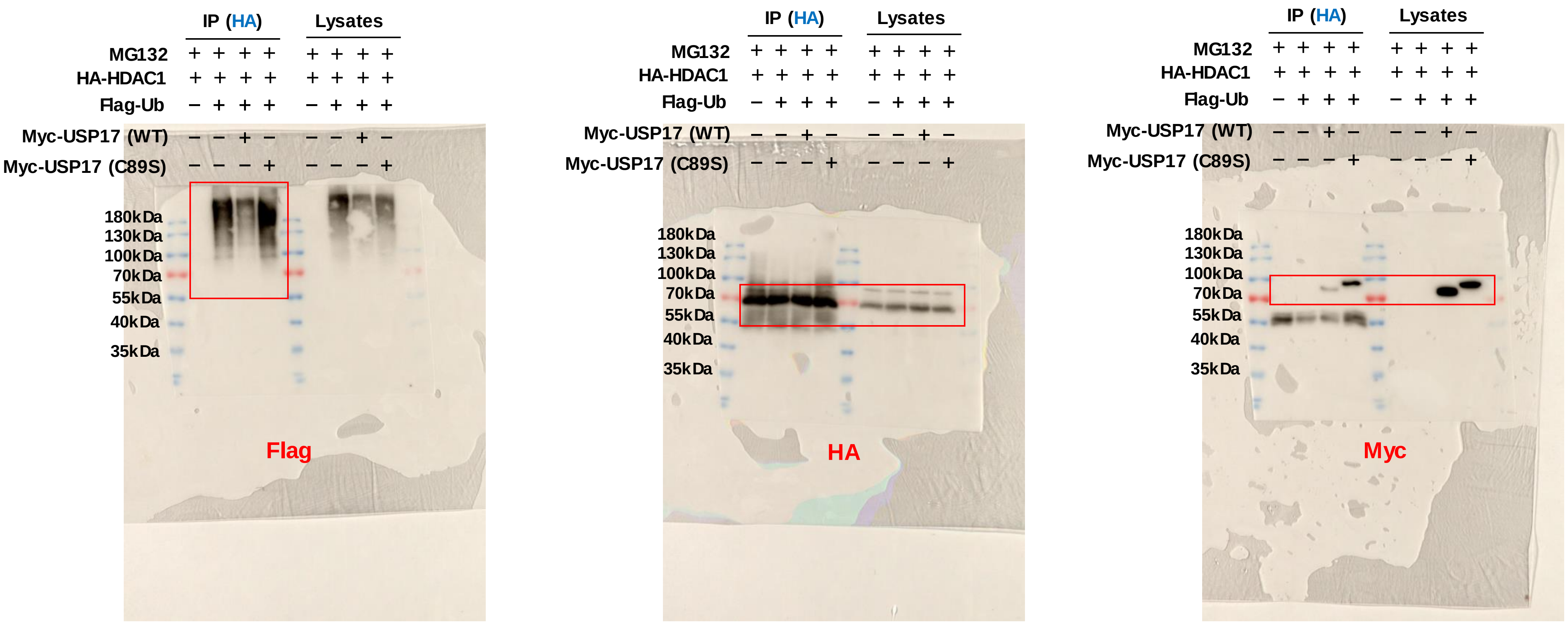

Figure S2E—validation of *USP17* overexpression

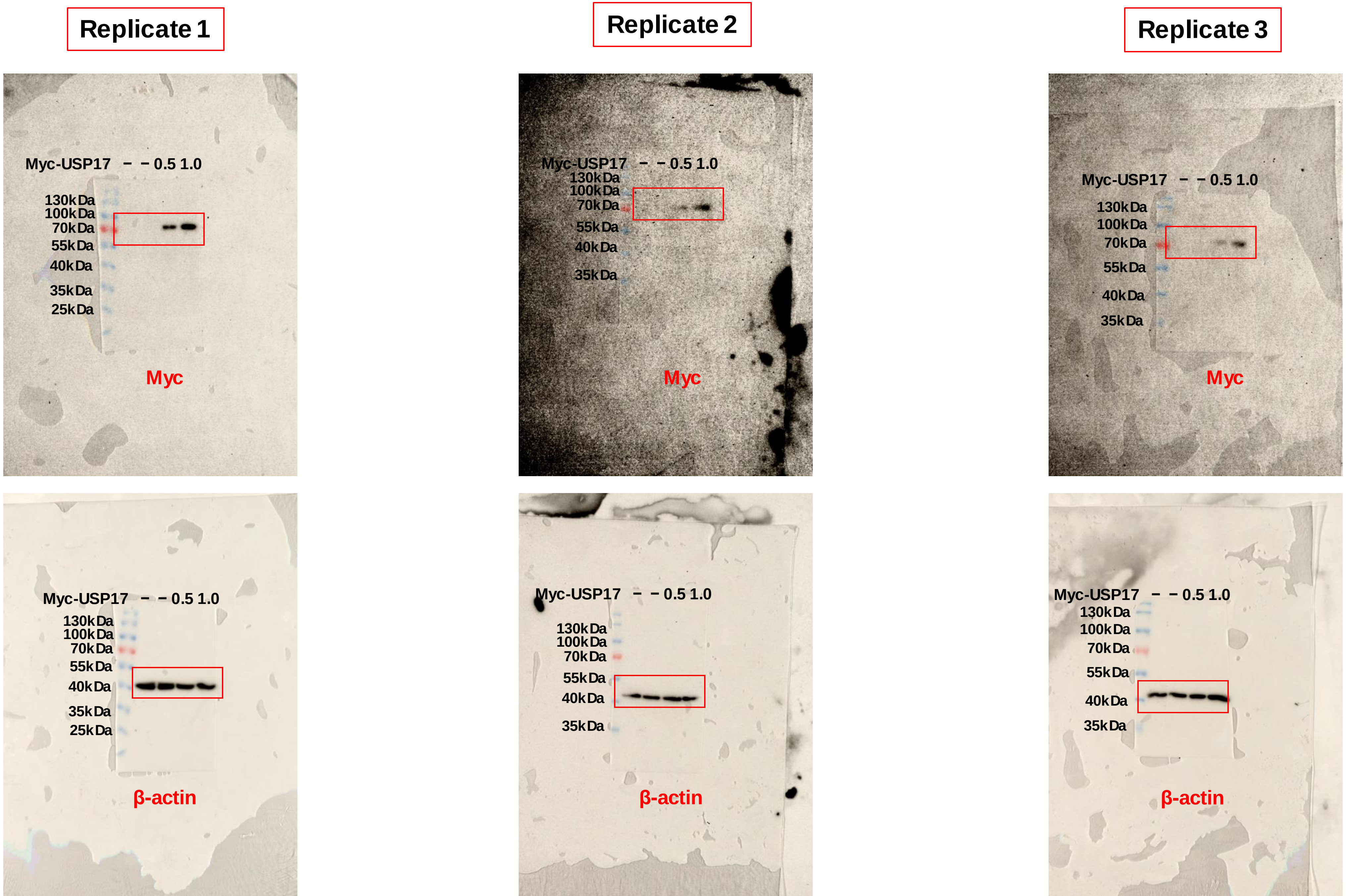

Figure S2G—The effect of *USP17* overexpression on 3T3-L1 adipocyte differentiation

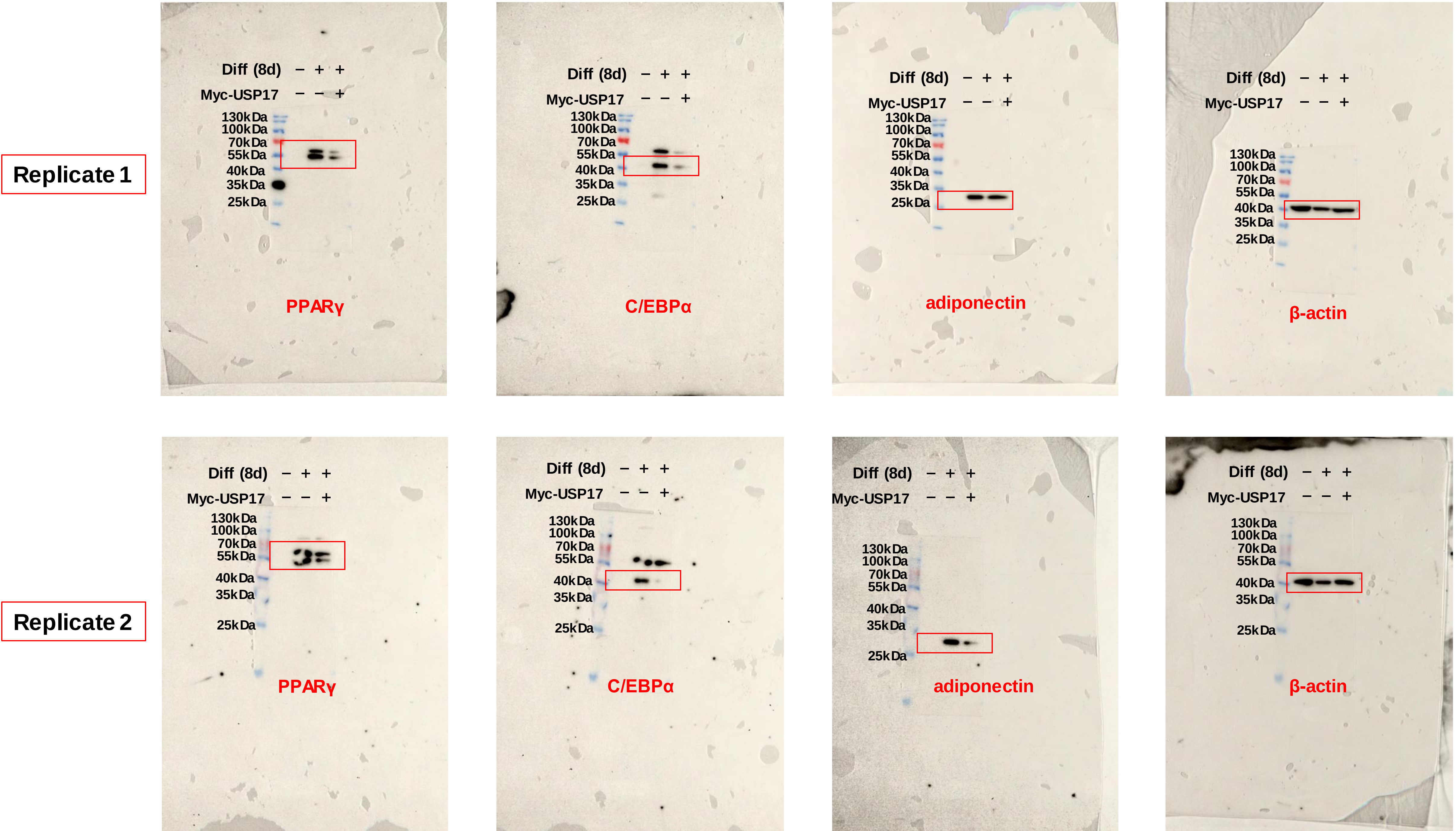

Figure S2G—The effect of *USP17* overexpression on 3T3-L1 adipocyte differentiation

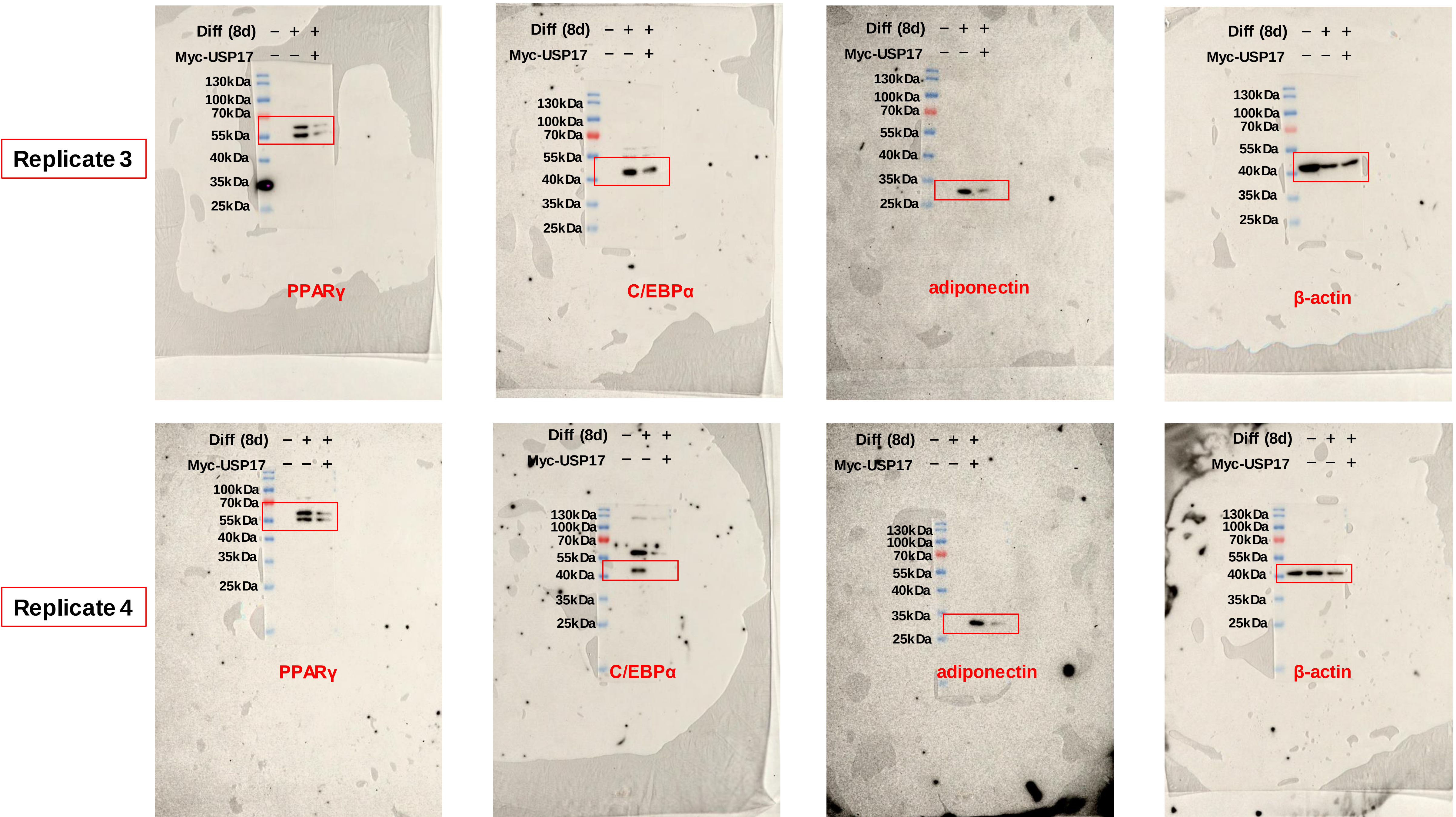

Figure S2G—The effect of *USP17* overexpression on 3T3-L1 adipocyte differentiation

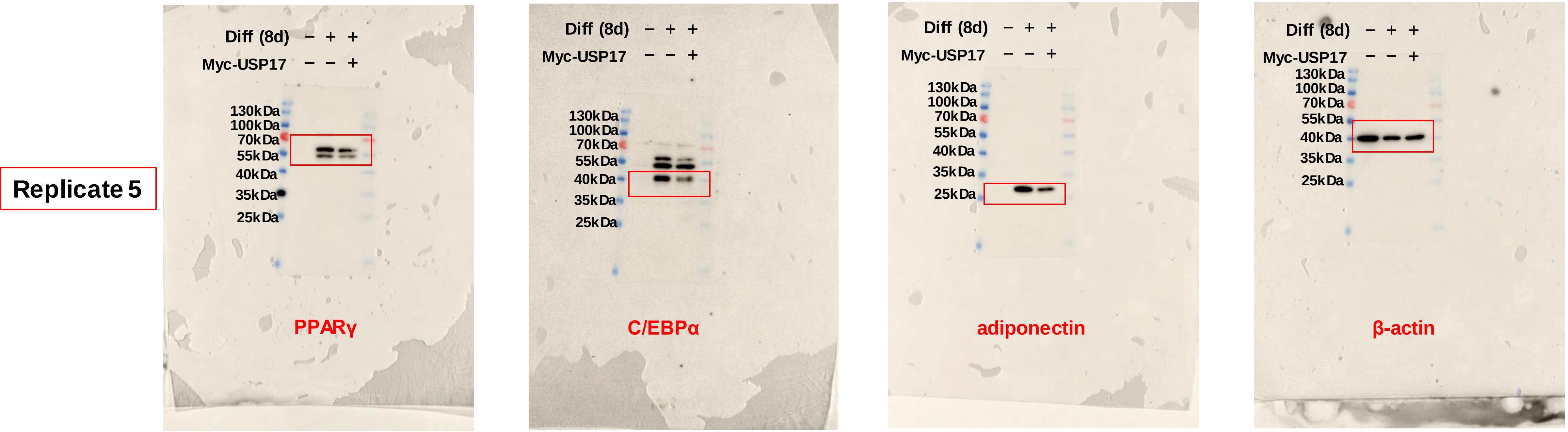

Figure S2J—validation of USP17 (WT) and USP17 (C89S) overexpression

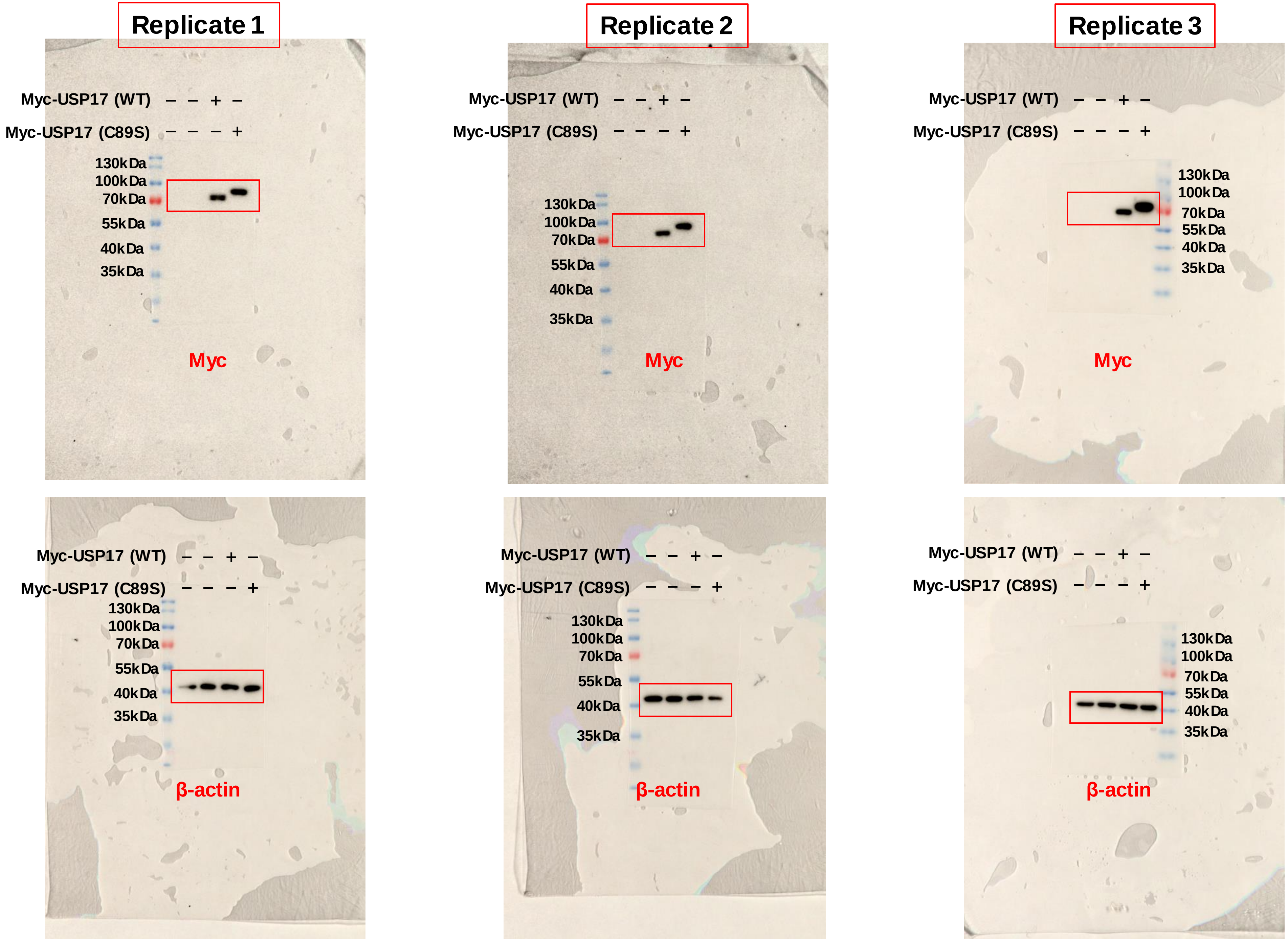

Figure S3A—The does effect of USP17 (WT) on HDAC1 stability

Replicate 1

Replicate 2

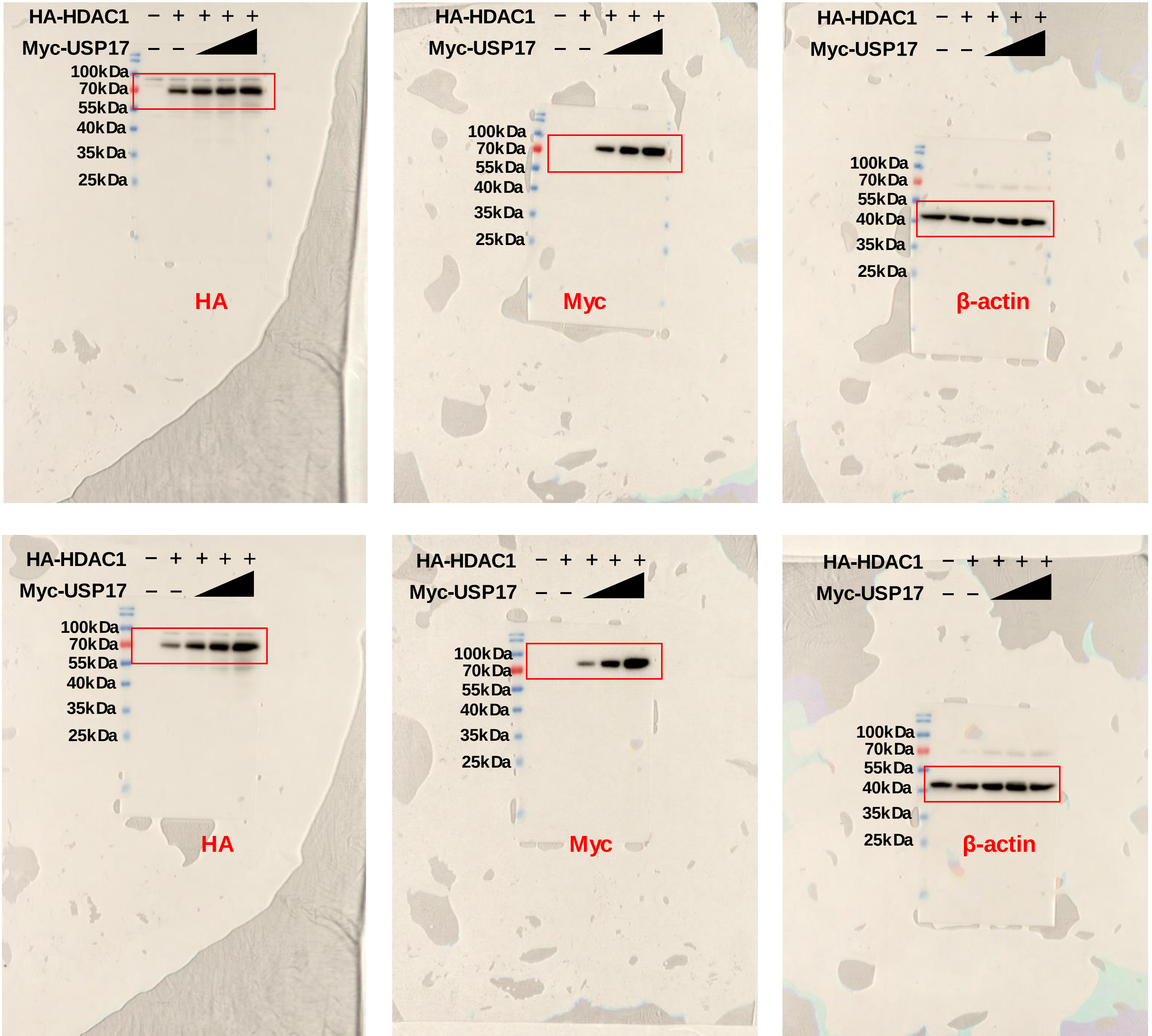

Figure S3A—The does effect of USP17 (WT) on HDAC1 stability

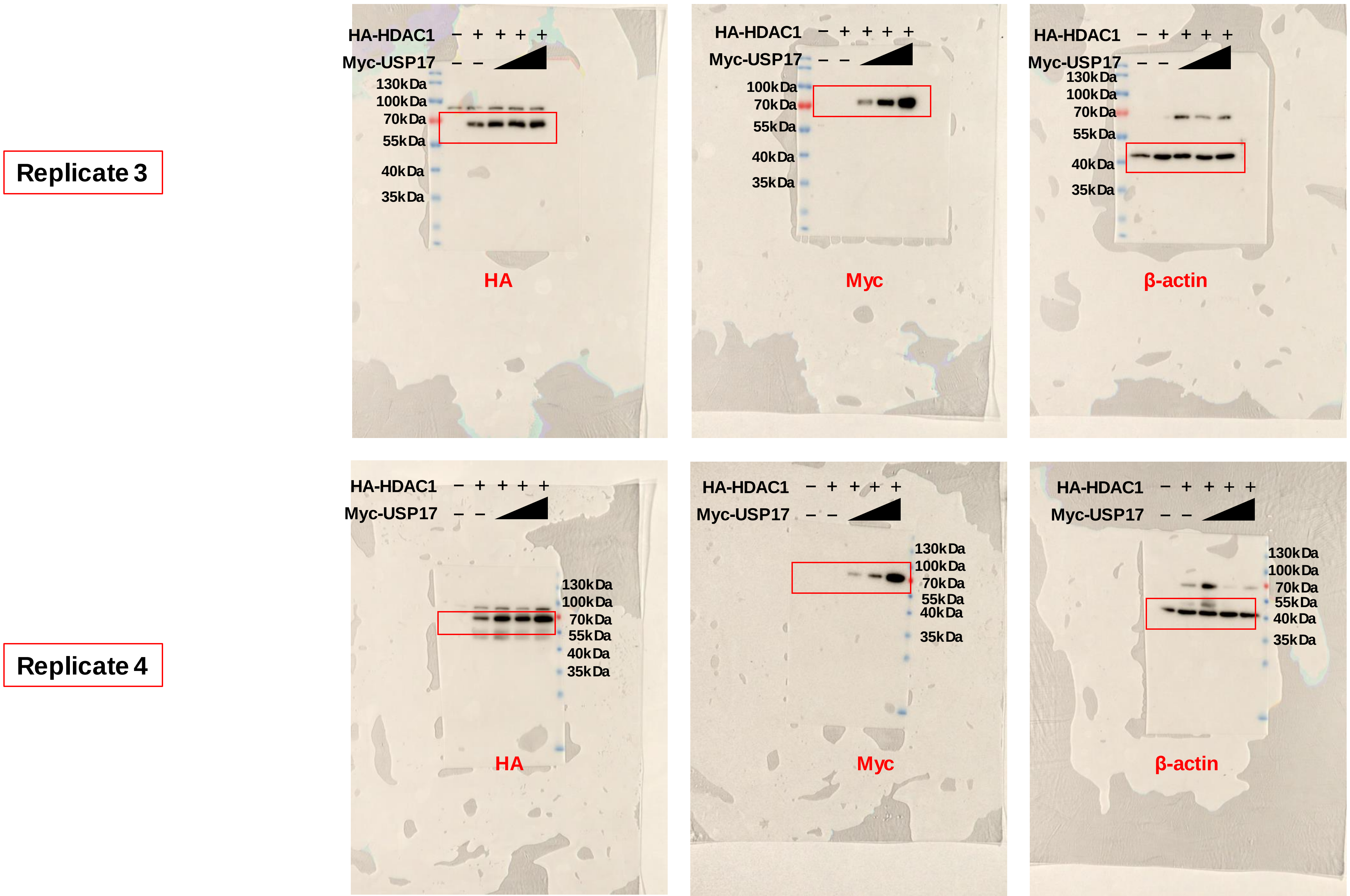

Supplement: Multimedia component 2 [file mmc2.pdf]
